# Functional Partitioning of Genomic Variance and Genome-Wide Association Study for Carcass Traits in Korean Hanwoo Cattle Using Imputed Sequence Level SNP Data

**DOI:** 10.3389/fgene.2018.00217

**Published:** 2018-06-22

**Authors:** Mohammad S. A. Bhuiyan, Dajeong Lim, Mina Park, Soohyun Lee, Yeongkuk Kim, Cedric Gondro, Byoungho Park, Seunghwan Lee

**Affiliations:** ^1^Department of Animal Science and Biotechnology, Chungnam National University, Daejeon, South Korea; ^2^Department of Animal Breeding and Genetics, Bangladesh Agricultural University, Mymensingh, Bangladesh; ^3^Division of Animal Genomics and Bioinformatics, National Institute of Animal Science, Rural Development Administration, Wanju, South Korea; ^4^Animal Genetic Improvement Division, National Institute of Animal Science, Rural Development Administration, Seonghwan, South Korea; ^5^College of Agriculture and Natural Resources, Michigan State University, East Lansing, MI, United States

**Keywords:** variance partitioning, genome level SNP, GWAS, carcass traits, Hanwoo cattle

## Abstract

Quantitative traits are usually controlled by numerous genomic variants with small individual effects, and variances associated with those traits are explained in a continuous manner. However, the relative contributions of genomic regions to observed genetic variations have not been well explored using sequence level single nucleotide polymorphism (SNP) information. Here, imputed sequence level SNP data (11,278,153 SNPs) of 2109 Hanwoo steers (Korean native cattle) were partitioned according to functional annotation, chromosome, and minor allele frequency (MAF). Genomic relationship matrices (GRMs) were constructed for each classified region and fitted in the model both separately and together for carcass weight (CWT), eye muscle area (EMA), backfat thickness (BFT), and marbling score (MS) traits. A genome-wide association study (GWAS) was performed to identify significantly associated variants in genic and exon regions using a linear mixed model, and the genetic contribution of each exonic SNP was determined using a Bayesian mixture model. Considering all SNPs together, the heritability estimates for CWT, EMA, BFT, and MS were 0.57 ± 0.05, 0.46 ± 0.05, 0.45 ± 0.05, and 0.49 ± 0.05, respectively, which reflected substantial genomic contributions. Joint analysis revealed that the variance explained by each chromosome was proportional to its physical length with weak linear relationships for all traits. Moreover, genomic variances explained by functional category and MAF class differed greatly among the traits studied in joint analysis. For example, exon regions had larger contributions for BFT (0.13 ± 0.08) and MS (0.22 ± 0.08), whereas intron and intergenic regions explained most of the total genomic variances for CWT and EMA (0.22 ± 0.09–0.32 ± 0.11). Considering different functional classes of exon regions and the per SNP contribution revealed the largest proportion of genetic variance was attributable to synonymous variants. GWAS detected 206 and 27 SNPs in genic and exon regions, respectively, on BTA4, BTA6, and BTA14 that were significantly associated with CWT and EMA. These SNPs were harbored by 31 candidate genes, among which *TOX, FAM184B, PPARGC1A, PRKDC, LCORL*, and *COL1A2* were noteworthy. BayesR analysis found that most SNPs (>93%) had very small effects and the 4.02–6.92% that had larger effects (10^-4^ × $\sigma_{A}^{2}$, 10^-3^ × $\sigma_{A}^{2}$, and 10^-2^ × $\sigma_{A}^{2}$) explained most of the total genetic variance, confirming polygenic components of the traits studied.

## Introduction

The genetic architecture of complex traits like carcass and meat quality in cattle includes a large number of loci with small individual effects on each trait. Variations in those traits are due to interactions among the loci dispersed across the genome as well as influenced by environmental factors. It is important to know how additive genetic variances are distributed across different genomic regions for better understanding of the genetic composition of complex traits. Several genome-wide association studies (GWAS) using dense single nucleotide polymorphism (SNP) marker panels have shown the differential contribution of genic and non-genic (intergenic) regions of genomes to additive genetic variance in human (Yang et al., 2011b; Lee et al., 2012), dairy and beef cattle (Koufariotis et al., 2014), and broiler chicken (Abdollahi-Arpanahi et al., 2016). These studies showed that genic regions usually contributed more additive genetic variation than non-genic regions. However, Santana et al. (2016) reported maximum genomic variance to be attributed to intergenic and intronic regions in beef cattle, whereas Do et al. (2015) found almost similar genomic contributions from annotated genic and non-genic regions in pigs. The differences among these studies might be associated with several factors, such as SNP density in the marker panel, statistical models used, species, and types of traits investigated.

The Encyclopedia of DNA Elements (ENCODE) project found that about 80% of the human genome was engaged in relevant biochemical activities, even though only about 1% of the genome encodes a defined product such as a protein or reproducible biochemical signature (ENCODE Project Consortium, 2012). Hindorff et al. (2009) reported that 88% of the total trait associated significant variants for human were located in intron (45%) and intergenic (43%) regions. But, importantly, SNPs in missense and promotor regions were significantly enriched whereas SNPs of intergenic regions were underrepresented in association studies (Hindorff et al., 2009; Kindt et al., 2013). On the other hand, the contribution of minor allele frequency (MAF) classes varied greatly for carcass traits in Japanese Black cattle (Ogawa et al., 2016) and for 17 different complex traits in Nordic Holstein cattle (Zhang et al., 2017). Therefore, understanding how genomic regions contribute to the variances of complex traits and partitioning the genome into different categories will help in describing a clear scenario of the genomic architecture of traits.

In GWAS, stringent statistical thresholds are considered in most cases to control false positive results using multiple hypothesis testing and, therefore, many variants with small effects fail to reach significance levels despite some of them being causal variants. The proportion of phenotypic variance explained by all SNPs is relatively lower than the estimates of pedigree data because the former includes only the contributions of causal variants that are in linkage disequilibrium (LD) with genotyped SNPs (Visscher et al., 2010). This is known as the perceived problem of “missing heritability” (Manolio et al., 2009). Insufficient LD between genotyped SNPs and causal variants accounts for most of the deviation in variance estimates. Lack of LD can also arise if the MAF of causal variants is lower than the genotyped SNPs (Lee et al., 2011). Imputation enables the determination of SNP genotypes that are not directly genotyped by low-density marker panels and uses information from a reference population that has been genotyped with higher-density SNP markers (Hickey et al., 2012). In GWAS, more causal variants of a given trait are expected to be detected using imputed whole-genome sequence data compared with the number of causal variants detected by the currently used SNP marker panels. In addition, LD between SNP markers and causal variants increases in association analysis from imputed sequence level SNP data, which also ensures higher reliability of genomic predictions for quantitative traits because more SNP information can be incorporated and genomically evaluated (van Binsbergen et al., 2015; Gonzalez-Pena et al., 2016). Therefore, sequence level SNP information can be used to capture the maximum numbers of attributed additive genetic variances in a whole-genome or a particular genomic region for better estimation of traits. Previous studies reported higher imputation accuracy from high density genotype to whole genome sequence variants which also provided better prediction for genomic selection in dairy and beef cattle (Hawlader et al., 2017; Pausch et al., 2017).

Hanwoo (*Bos taurus coreanae*), an indigenous cattle breed of South Korea, has been bred intensively over the last four decades for the improvement of carcass and meat quality traits. Hanwoo beef is regarded as a cultural icon and is very popular for its extensive marbling and eating quality attributes like tenderness, juiciness, and characteristic flavor (Jo et al., 2012). Presently, the genetic worth of individual Hanwoo is estimated based on carcass weight (CWT), eye muscle area (EMA), backfat thickness (BT), and marbling score (MS) traits using both pedigree and SNP genotype data (Lee et al., 2014). Previous GWAS using a 50-K SNP marker panel detected a number of significant SNPs associated with CWT, intramuscular fat, Warner–Bratzler shear force, and sensory traits in Hanwoo (Lee et al., 2013, 2014; Dang et al., 2014). Notably, the genetic evaluation of complex traits using genomic information is increasingly being used in different cattle breeding programs. However, until now, GWAS or genetic architecture of carcass and meat quality traits using sequence level SNP information has been limited to other beef cattle breeds and has not yet been reported in Hanwoo cattle. In this study, imputed genome sequence level SNP data were used to investigate genetic variance explained by subsets of genomic regions as well as to identify genomic variants in genic and exon regions and their contributions by GWAS for four carcass and meat quality traits in Hanwoo cattle.

## Materials and Methods

### Animals and Phenotypes

A total of 2109 Hanwoo steers born between 2004 and 2013 at Hanwoo Experiment Station, National Institute of Animal Science (NIAS), Rural Development Administration, South Korea, were used in this study. All the steers were progeny of 251 sires and unrelated dams (1–3 progenies per dam). Animal health and welfare issues were followed according to approved guidelines of the Animal Care and Use Committee (NIAS) and the ethics committee approval number was 2015-150. Feeding and management practices were uniform under feedlot condition with a concentrate mixture and rice straw-based ration. In the total feed, the proportions of concentrate and roughage were approximately 1.5:1, 2.5:1, and 4.5:1 in the grower (4–12 months), fattening I (13–18 months), and fattening II (19–23 months) rations, respectively. Crude protein and total digestible nutrients contents in the concentrate mixtures of these three rations were 14–16 and 68–70%, 11–13 and 71–73, and 11–12 and 72–73%, respectively. All animals were slaughtered at about 24 months of age. The carcass and meat quality traits investigated in this study were CWT, EMA, BFT, and MS. Feeding, management, and trait measurements were according to Bhuiyan et al. (2017). Briefly, the cold CWT was taken after chilling for about 24 h. *Longissimus dorsi* muscle samples (approximately 1.5 kg) were collected from the junction between the 12th and 13th rib for the EMA, MS, and BFT measurements. MS was assessed on a 1–9 point scale according to the Korean Beef Marbling Standard (KAPE, 2012). Descriptive statistics of carcass and meat-quality traits are summarized in Supplementary Table S1.

### SNP Genotyping and Quality Control

In total, 2605 individuals were genotyped initially using two different SNP platforms, Illumina Bovine SNP50 BeadChip (1677 animals) and Bovine HD BeadChip (928 animals). The unphased genotypes were converted into phased data using Eagle v. 2.3.2 based on long-range phasing approach (Loh et al., 2016). The genotype data for all 1677 individuals were then imputed to a high-density level (671,902 SNPs) considering the high-density genotype data as reference sequence panel using Minimac3 (Das et al., 2016). SNPs on the sex chromosomes were excluded. Whole-genome sequence data of 203 progeny tested Hanwoo bulls (South Korea Proven Bulls) were used as the reference population for sequence level SNP imputation. Finally, high-density genotypes of 2109 Hanwoo steers were imputed one chromosome at a time to sequence level using Minimac3, where each sequenced individual had 25,676,502 SNPs. We set-up imputation *R^2^* > 0.60 according to a previous Cross-Disorder Group of the Psychiatric Genomics Consortium et al. (2013) study, which included 49.12% of the total imputed SNPs. SNP filtering was performed based on the following exclusion criteria: MAF < 0.01 and Hardy–Weinberg equilibrium <0.0001 using PLINK 1.9 software (Purcell et al., 2007). After quality control, 11,278,153 SNPs were retained for further analyses.

### SNP Annotation

The physical positions of the imputed SNPs were determined using the UMD 3.1 (Elsik et al., 2016) bovine genome assembly as a reference sequence. SNP annotation, filtering, and partitioning were performed using SnpEff v.4.3p (Cingolani et al., 2012b) and SnpSift software (Cingolani et al., 2012a). Total SNPs were partitioned into 14 different categories according to their functional annotations (**Table 1**) except regulatory regions. Then, all splice variants and start and stop sites were excluded because they contained a very low proportion of the total SNPs or because, in exon regions, SNPs might already be represented by coding sequences and untranslated regions (UTRs). Finally, six major functional classes of genomic regions were considered: synonymous, non-synonymous (missense), 5′- and 3′-UTRs, intron, regulatory, and intergenic regions. Regulatory regions were defined as regions located 5-kb upstream and 5-kb downstream of genes, and intergenic regions were defined as regions more than 5-kb distant from genes. Besides, the variants were categorized into six classes based on their MAF as 0.01–0.05, 0.05–0.1, 0.1–0.2, 0.2–0.3, 0.3–0.4, and 0.4–0.5.

**Table 1:** Number of variants annotated in different functional classes in Korean Hanwoo cattle using sequence level single nucleotide polymorphism (SNP) data^1^.

Functional class	Number of variants	Proportion (%)
Intergenic region	7,928,883	70.303
Intron variant	3,246,727	28.788
Exon variant	99,204	0.880
Synonymous variant	50,040	0.444
Missense variant	21,064	0.187
Downstream gene variant	469,605	4.164
Upstream gene variant	460,915	4.087
5′ UTR variant	5,119	0.045
3′ UTR variant	24,773	0.220
Splice region variant	8,903	0.079
All stop variants	226	0.002
Splice acceptor variant	184	0.002
Splice donor variant	177	0.002
Start lost variant	26	0.000

*^*1*^Functional annotation of SNP variants was performed based on the cattle genome reference sequence (UMD 3.1) using SnpEff ver. 4.3p and SnpSift software (Cingolani et al., 2012a,b). The cumulative value is higher than 100% as some variants are located in several transcripts and therefore, could be allocated to multiple regions.*

### Genomic Variance Partitioning

To decipher the genomic architecture of traits and predictive ability of particular genomic regions, the total genomic variance was partitioned based on MAF category (six classes), chromosome (29 autosome), and functional annotations (six classes). To do this, genomic relationship matrices (GRMs) were estimated based on the SNPs in the respective categories (MAF, chromosome, and functional class) following the method of VanRaden (2008) using genome-wide complex trait analysis (GCTA v.1.26) software (Yang et al., 2011a). The variance attributable to each category was calculated separately or by fitting all GRMs of the respective category simultaneously in a joint analysis. Restricted maximum likelihood analysis implemented in GCTA v.1.26 was performed using the following linear mixed model:

$$y=X\beta +\sum_{G=1}^{n}g_{G}+e\;$$

where *y* is the vector of phenotypes, β is a vector of fixed effects (year and season) and covariate (age) with its incidence matrix *X, n* is the number of subsets for non-overlapping SNPs partitioning (*n* = 6 for joint analysis by MAF bin, *n* = 29 for the number of autosomes, and *n* = 6 for the functional annotation of SNPs), *g*_G_ is a vector of random additive genetic effects attributed from aggregated SNP information, and *e* is a random residual error. The variance component of phenotypic values from the joint analysis is $V_{\text{g}}\text{=}A_{\text{g}}\sigma_{\text{g}}^{\text{2}}\text{+}I\;\sigma_{\text{e}}^{\text{2}}$, where $\sigma_{g}^{2}$ is the additive genetic variance tagged by SNPs, A_g_ is the genetic relationship matrix calculated from SNP data, $\sigma_{e}^{2}$ is the error variance, and I is the identity matrix. The proportion of variance captured by each category is calculated as $h_{\text{G}}^{2}=\sigma_{\text{G}}^{2}/\sigma_{\text{P}}^{2}$, where $\sigma_{P}^{2}$ denotes the phenotypic variance explained by all autosomal SNPs.

### Genome-Wide Association and Genetic Contribution of SNPs

Two different approaches were used for the single-marker association analysis using SNPs in genic (exon or intronic SNPs) and exon regions, as well as to know the contribution of exonic SNPs to phenotypes. Phenotypic data were adjusted using a linear mixed model for fixed effects (year and season) and covariate (animal’s age at slaughter). The adjusted phenotypes and constructed GRMs were subsequently used for GWAS under a mixed linear model including all candidate SNPs implemented in GCTA v.1.26. In GCTA, the mixed linear model assumes that all markers are to be in LD with quantitative trait loci (QTL) in close proximity and additive effects are derived based on the SNP mediated overall covariance. Thus, single trait association analysis was performed using the following model:

y=a+bX+g+e

where *y* is the adjusted phenotypic value, *a* is the mean, *b* is the additive effect (fixed effect) of the candidate SNP to be tested for association, *X* is the SNP genotype indicator variable coded as 0, 1, or 2 depending on the number of copies of a specified allele, *g* is the accumulated effect of all SNPs, and *e* is the random residual effect. The Bonferroni adjusted *P*-value threshold was determined to correct multiple hypotheses testing at the genome-wide suggestive (1.0/number of SNPs tested) and significant (0.05/number of SNPs tested) levels. Manhattan plots were drawn from genome-wide associated *P*-values (-log10 transformed observed *P*-values) using the “gap” package (Zhao, 2014) in R program. A Bayesian mixture model implemented in BayesR software^1^ that fitted all markers simultaneously with four posterior distributions of each marker was used to estimate the variance explained by exonic SNPs. The SNPs in the mixture model were assumed to be normally distributed with the proportion of effect sizes 0.00, 0.0001, 0.001, and 0.01, using a single chain length of 50,000 samples, where the first 20,000 cycles were discarded as burn-in (Erbe et al., 2012). The percentage of genetic contribution (%*V*_g_) accounted for by each SNP was calculated using the formula:

$$\%V_{\text{g}}=100\times \frac{2pq\beta^{2}}{\sigma_{\text{A}}^{2}}$$

where, *p* and *q* are the allele frequencies for a given trait, β is the additive effects of the SNPs, and $\sigma_{A}^{2}$ is the additive genetic variance for a trait. Besides, the per SNP based genetic variance explained by each annotated class was estimated according to the methods described by Koufariotis et al. (2014) using following formula:

$$VarPerSNP=\frac{\left[(h^{2}÷n)\times 100\right]}{10^{-4}}$$

where, *h*^2^is the heritability, *n* is the total SNPs in the respective annotated class, results were multiplied by 100 to get percent (%) of the genetic variance explained and results were divided by 10^-4^ for visualization of the data. The derived variance components ($\sigma_{A}^{2}$ and $\sigma_{P}^{2}$) during individual SNP effect calculation were used for *h*^2^ estimates. Subsequently, we performed functional annotation of the significant SNPs and searched for candidate genes using SnpEff v.4.3q and variant effect predictor (VEP) tools supported by Ensembl (McLaren et al., 2016).

## Results

### Annotation and Distribution of Variants Across the Genome

Genome sequence level SNP data were annotated into 14 different functional classes (**Table 1**) However, because of the low SNP proportion in some classes, only six major classes (synonymous, non-synonymous, 5′- and 3′-UTRs, intron, regulatory, and intergenic regions) were included in our analysis. As expected, intergenic variants were the most common, followed by intron, upstream and downstream, and exon variants, representing 70.30, 28.79, 8.24, and 0.88% of the total SNPs, respectively. The proportions of SNPs in the other functional categories were very low (0.002–0.22% of the total SNPs). In a previous study using bovine next-generation sequencing data, Aßmus et al. (2011) found almost similar proportions of intron (28.04%) and exon variants (0.90%) in cattle; however, they reported relatively lower proportions of intergenic (64.36%) and regulatory region (6.38%) variants. Our results are close to the findings of Koufariotis et al. (2014) who reported the proportion of SNPs based on 777-K data in the aforementioned four classes to be 67.0, 31.0, 8.0, and 1.0%, respectively, in beef cattle. Santana et al. (2016) reported the distribution of SNPs in intergenic, intron, and exon regions was 63.64, 28.17, and 1.46%, respectively, in Nellore cattle, which also supports our results. Taken together, the results indicate that several attributes like SNP density, LD among SNPs, poor functional annotation, and types of traits may affect the annotation results.

### Partition of Genomic Variance Explained by Individual Chromosomes

**FIGURE 1:**
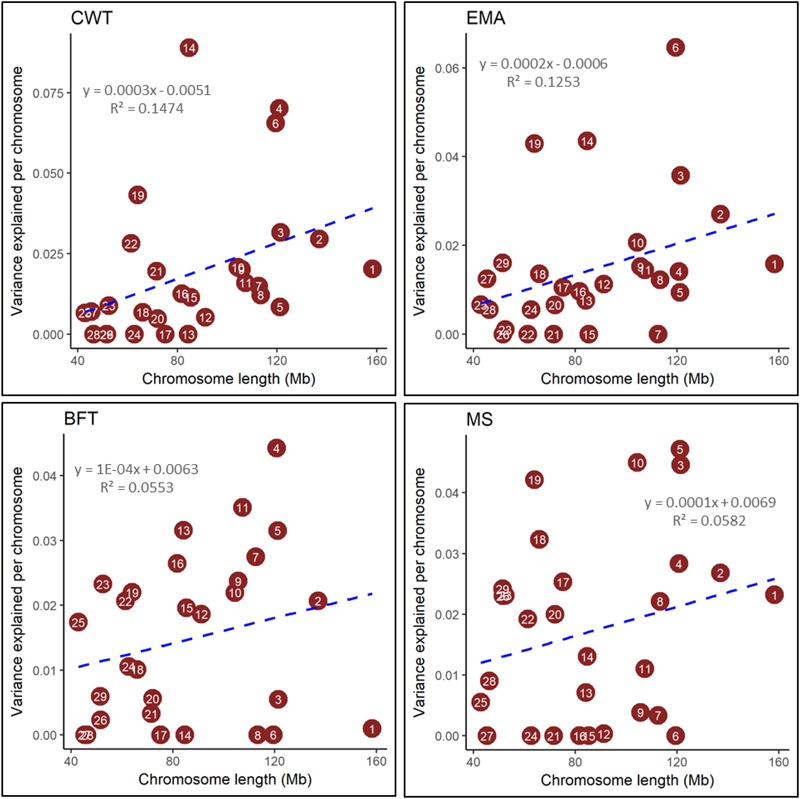
Estimated proportion of variance explained by each chromosome for carcass weight (CWT), eye muscle area (EMA), backfat thickness (BFT), and marbling score (MS) against its length. Genomic partitioning was performed by joint analysis. The number in the circles represent the chromosome number.

The proportions of genomic variance attributed to all SNPs were found to be 0.57, 0.44, 0.45, and 0.49 for CWT, EMA, BFT, and MS, respectively (**Table 2**), suggesting that a substantial genomic contribution explained the phenotypic variation in the studied population. To determine what proportions of the variance were explained by individual chromosomes, we performed a joint analysis by fitting 29 GRMs (from 29 autosomes) simultaneously. The chromosomes contributed to the total genomic variance in various degrees; namely, from 0.000 to 0.089 for CWT, from 0.000 to 0.064 for EMA, from 0.000 to 0.044 for BFT, and from 0.000 to 0.047 for MS. Moreover, the sum of variances attributed to individual chromosomes was slightly lower than the estimated total genomic variance for all four traits (Supplementary Table S2). Notably, with few exceptions, the amount of variance explained by each chromosome was found to be proportional to its physical length for all four traits (**Figure 1**). However, the magnitudes of linear relationships (*R^2^*) were comparatively low and varied from 0.06 to 0.15 among the four traits studied.

**Table 2:** Estimates of the variance explained by the SNPs located in exon, intron, and intergenic regions for four carcass and meat quality traits in Korean Hanwoo cattle.

Category	Number of SNPs	Calculation method^∗^	*h^2^* (S.E.)			
			CWT	EMA	BFT	MS
Exon	99204	Separate	0.45 (0.04)	0.34 (0.04)	0.38 (0.04)	0.43 (0.04)
		Joint	0.0001(0.07)	0.0001(0.08)	0.13 (0.08)	0.22 (0.08)
Intron	3246727	Separate	0.53 (0.05)	0.41 (0.05)	0.41 (0.05)	0.46 (0.05)
		Joint	0.32 (0.11)	0.24 (0.11)	0.13 (0.10)	0.09 (0.11)
Intergenic	7928883	Separate	0.55 (0.05)	0.43 (0.05)	0.43 (0.05)	0.46 (0.05)
		Joint	0.25 (0.09)	0.22 (0.09)	0.19 (0.10)	0.18 (0.09)
Total	11278153	Separate	0.57 (0.05)	0.44 (0.05)	0.45 (0.05)	0.49 (0.05)
		Joint	0.57 (0.05)	0.45 (0.05)	0.45 (0.05)	0.49 (0.05)

*^∗^Separate means individual analysis was performed for each trait considering the SNPs of respective functional annotation, joint means all three categories (exon, intron, and intergenic) were considered in a single analysis, values in the parentheses denote standard error of h^*2*^ estimates, CWT, carcass weight; EMA, eye muscle area; BFT, backfat thickness; MS, marbling score*.

### Partition of Genomic Variance Explained by Functional Annotation

To determine the genomic variation that was explained by the six major functional classes, at first, similarity matrices of each category were used separately and then all the matrices were fitted simultaneously in a joint analysis. The separate analyses showed that the six classes explained substantial amounts of the genomic variations for all traits, and their contributions were larger than those from the joint analysis (**Tables 2, 3**). For the separate analyses, the LD between SNPs in the different functional classes might have led to overestimation of the genomic variance for each class. For the joint analysis, the genomic variances explained by genic (synonymous, non-synonymous, and 5′- and 3′-UTRs) and upstream and downstream regulatory variants were negligible and close to zero (data not shown) for the four traits studied. Therefore, the variants in those functional classes were merged with the exon and intergenic classes, respectively. The sum of variances for both the functional classes and the MAF categories were similar to the estimates for the separate and joint analyses using all the SNPs (**Tables 2, 4**) for all four traits and justified the well-fitted genome partitioning analysis.

**Table 3:** Estimated proportion of variance explained by the synonymous, non-synonymous, and 5′–3′ UTR SNPs for four carcass and meat quality traits^1^.

Category	Number of SNPs	Calculation method^∗^	*h^2^* (S.E.)			
			CWT	EMA	BFT	MS
5′–3′ UTR	28100	Separate	0.37 (0.04)	0.30 (0.04)	0.31 (0.04)	0.36 (0.04)
		Joint	0.09 (0.06)	0.10 (0.06)	0.04 (0.06)	0.09 (0.06)
Synonymous	50040	Separate	0.44 (0.04)	0.32 (0.04)	0.38 (0.04)	0.42 (0.04)
		Joint	0.30 (0.09)	0.22 (0.09)	0.30 (0.09)	0.29 (0.09)
Non-synonymous	21064	Separate	0.38 (0.04)	0.28 (0.04)	0.33 (0.04)	0.37 (0.04)
		Joint	0.06 (0.07)	0.02 (0.07)	0.04 (0.07)	0.05 (0.07)
Total	99204	Joint	0.45 (0.04)	0.34 (0.04)	0.38 (0.04)	0.43 (0.04)

*^∗^SNPs in exon regions were analyzed either separately for each functional category (synonymous, non-synonymous, and UTR) or jointly in a single analysis. ^*1*^See **Table 2** for trait abbreviations*.

**Table 4:** Estimated proportion of variance explained by different minor allele frequency (MAF) category for four carcass and meat quality traits in Korean Hanwoo cattle^1^.

MAF of SNPs	Number of SNPs	Calculation method^∗^	*h^2^* (S.E.)			
			CWT	EMA	BFT	MS
0.01–0.05	3151789	Separate	0.39 (0.04)	0.32 (0.04)	0.36 (0.04)	0.39 (0.04)
	[0.279]	Joint	0.06 (0.05)	0.02 (0.05)	0.15 (0.06)	0.08 (0.06)
0.05–0.1	1661170	Separate	0.43 (0.04)	0.42 (0.05)	0.36 (0.04)	0.40 (0.04)
	[0.147]	Joint	0.01 (0.06)	0.09 (0.07)	0.00001(0.06)	0.06 (0.07)
0.1–0.2	2079582	Separate	0.53 (0.05)	0.42 (0.05)	0.39 (0.05)	0.44 (0.05)
	[0.184]	Joint	0.26 (0.09)	0.14 (0.09)	0.09 (0.09)	0.12 (0.10)
0.2–0.3	1603981	Separate	0.51 (0.05)	0.40 (0.04)	0.40 (0.05)	0.42 (0.05)
	[0.142]	Joint	0.10 (0.09)	0.02 (0.09)	0.07 (0.10)	0.00001(0.10)
0.3–0.4	1421961	Separate	0.51 (0.05)	0.39 (0.04)	0.39 (0.04)	0.44 (0.04)
	[0.126]	Joint	0.10 (0.09)	0.10 (0.10)	0.14 (0.10)	0.23 (0.10)
0.4–0.5	1359670	Separate	0.48 (0.04)	0.39 (0.04)	0.36 (0.04)	0.40 (0.04)
	[0.121]	Joint	0.05 (0.08)	0.07 (0.08)	0.00001(0.08)	0.002 (0.09)
Total	11278153	Separate	0.57 (0.05)	0.44 (0.05)	0.45 (0.05)	0.49 (0.05)
		Joint	0.58 (0.05)	0.44 (0.05)	0.45 (0.05)	0.49 (0.05)

*^∗^Separate means five analysis were performed separately for traits under each MAF bin, joint means all five MAF categories were considered in a single analysis, values in the parentheses denote standard error of h^*2*^ estimates, values in the square brackets represent the proportion of SNPs in each MAF category. ^*1*^See **Table 2** for trait abbreviations*.

**FIGURE 2:**
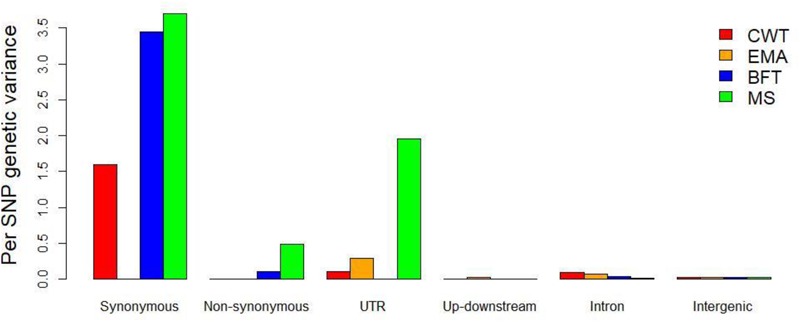
Estimated proportion of genetic variance explained by single nucleotide polymorphism (SNP) under each functional class when fitted jointly in the model. The genetic variance attributed with each SNP is expressed as percentage.

In the joint analysis, the genomic variances that accounted for the six functional classes varied among the carcass and meat quality traits. For example, the genomic heritability explained by exons was 0.13 and 0.22 for the BFT and MS traits, respectively, but close to zero for the CWT and EMA traits, whereas the genomic heritability explained by intron and intergenic regions ranged from 0.22 to 0.32 for the CWT and EMA traits, and from 0.09 to 0.19 for the BFT and MS traits. These results suggest distinct genetic architectures underlie the processes involved in muscle development and fat biosynthesis in the studied population. In particular, when the different functional classes in the exon regions (5′- and 3′-UTRs, synonymous and non-synonymous) were considered in the joint analysis, the genomic variances attributable to the synonymous class were significantly more than those attributable to the 5′- and 3′-UTRs and non-synonymous classes for all four traits. In the joint analysis, the genetic variance explained by each SNP was estimated to determine the contribution of the SNPs in each class. Regardless of the trait studied, the per SNP analysis also revealed that the variants in coding and UTR regions contributed more to the variance than variants in the intron and intergenic regions. Specifically, the largest proportion of the genetic variance was explained per SNP in the synonymous class, particularly for the CWT, BFT, and MS traits (**Figure 2**). Relatively lower genetic variance was explained per SNP in the UTRs for the CWT, EMA, and MS traits, and by SNPs in the non-synonymous class for the BFT and MS traits. In the intron class, the genetic variance explained per SNP was low, but higher than that for the upstream and downstream and intergenic classes for all four traits.

### Partition of Genomic Variance Explained by MAF Class

The distribution of SNPs in the six different MAF classes was 27.90, 14.70, 18.40, 14.20, 12.60, and 12.10% of the total SNPs (**Table 4**). Similar to the results for the functional annotations, the variance explained by the six different MAF bins from a joint analysis varied greatly among the traits and MAF categories. In general, two common alleles groups (0.10–0.20 and 0.30–0.40) contributed more to the variance for all traits than the other allele groups. Specifically, the highest genomic variance was explained by SNPs in MAF category 0.10–0.20 for the CWT (0.26) and EMA (0.14) traits, and by SNPs in MAF category 0.30–0.40 for the MS (0.23) and BFT (0.14) traits. Remarkably, the low frequent alleles (MAF < 0.05) accounted for the highest variance only for the BFT (0.15) trait. The other three MAF bins explained comparatively lower proportions of the genetic variance (from close to zero to 0.10) for all four traits investigated. This finding supports the idea that different genomic architectures exist between carcass and meat quality traits in Hanwoo cattle.

### Identification of Genomic Variants Through GWAS

**FIGURE 3:**
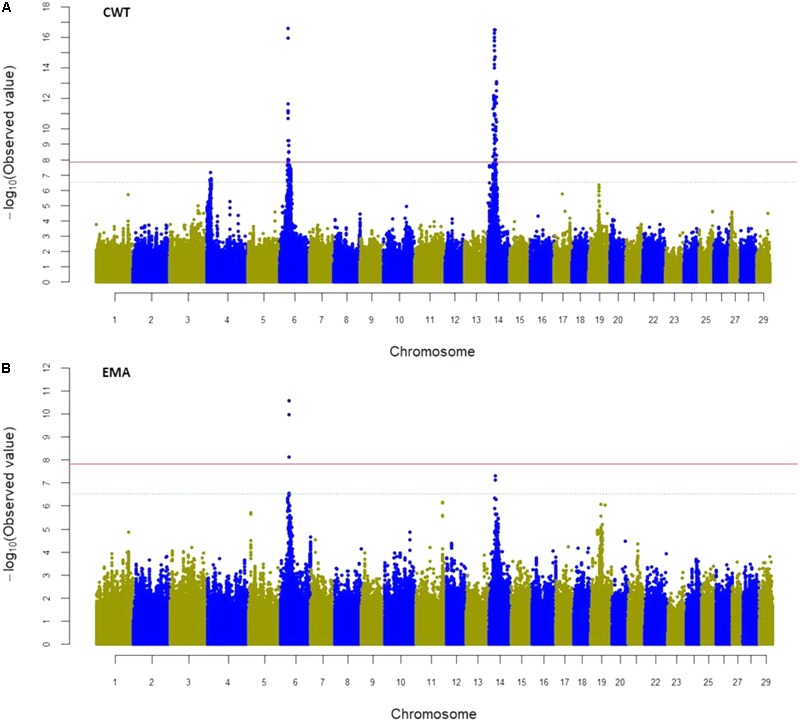
Manhattan plot of genome-wide association study (GWAS) using SNPs of genic (exon and intron) regions for CWT **(A)** and EMA **(B)** traits where *Y*-axis defines -log10 (*P*)-value against their respective positions on each chromosome (*X*-axis). The horizontal solid and dot lines indicate the Bonferroni adjusted significant (*P* < 1.49 × 10^-8^) and suggestive (*P* < 2.99 × 10^-7^) thresholds level, respectively.

Genome-wide association study was performed using SNPs in both genic (exon and intron together) and exon regions to identify their intra-genetic association with the four traits studied. Considering all the SNPs in the genic region (a total of 3,345,931 SNPs), the mixed linear model-based GWAS revealed 206 SNPs significantly associated with CWT (*P* < 1.49 × 10^-8^) and six SNPs significantly associated with EMA. These significant SNPs were located on BTA6 and 14, and were harbored by 24 candidate genes (**Figure 3, Table 5**, and Supplementary Table S3). The most significant SNPs (rs109438687 and rs109467519) were located in the introns of *FAM184B* on BTA6 and were associated with CWT. The top seven intronic SNPs were in *TOX* on BTA14 (rs41724548, rs41724547, rs41724546, rs42406058, rs42406039, rs109374728, and rs41724619) and had the second highest association with CWT. Significant SNPs for the CWT and EMA traits were located at 3.32 Mb on BTA6 and were in *LAP3, FAM184B, NCAPG, LCORL*, and *SLIT2*. Besides, significantly associated SNPs for CWT spanned a 13.69 Mb region on BTA14 that harbored 19 genes, among which *PRKDC, XKR4, IMPAD1, SDCBP, TOX, DNAJC5B, PREX2, C8orf46*, and *C8orf34* were notable (**Table 5** and Supplementary Table S3). These results indicate that these two regions of BTA6 and BTA14 were potential candidates for carcass traits in Hanwoo cattle. However, none of the SNPs reached significant levels for the BFT and MS traits (Supplementary Figure S1).

**Table 5:** Significant genic SNPs harbored genes for CWT and EMA traits in Korean Hanwoo cattle.

Ensembl ID^1^	Gene symbol	BTA^2^	Position^3^ (bp)	No. of SNPs	*P*-value^4^
ENSBTAG00000005989	*LAP3*	6	38577764 ~ 38583582	6	1.22E-08
ENSBTAG00000005932	*FAM184B*	6	38648218 ~ 38670165	5	1.06E-16
ENSBTAG00000021582	*NCAPG*	6	38794618 ~ 38804348	5	1.35E-08
ENSBTAG00000046561	*LCORL*	6	38849296 ~ 38900113	11	1.35E-08
ENSBTAG00000005108	*SLIT2*	6	41262050 ~ 41526051	10	1.02E-08
ENSBTAG00000047743	*KCNIP4*	6	41845249 ~ 41900486	2	2.84E-09
ENSBTAG00000044106	*SPIDR*	14	20753321 ~ 20875591	5	5.95E-09
ENSBTAG00000017019	*PRKDC*	14	21043161 ~ 21151156	32	1.15E-12
ENSBTAG00000044050	*XKR4*	14	24332803 ~ 24474674	8	1.36E-08
ENSBTAG00000015637	*IMPAD1*	14	25546508 ~ 25560744	6	1.29E-11
ENSBTAG00000005287	*CYP7A1*	14	26351959 ~ 26351959	1	2.82E-15
ENSBTAG00000019910	*SDCBP*	14	26443481 ~ 26445603	2	5.75E-15
ENSBTAG00000008958	*NSMAF*	14	26496858 ~ 26496858	1	3.62E-16
ENSBTAG00000004954	*TOX*	14	26631471 ~ 26941314	48	1.25E-08
ENSBTAG00000026283	*ASPH*	14	28702223 ~ 28712341	6	1.09E-08
ENSBTAG00000001299	*CYP7B1*	14	31058827 ~ 31064078	3	1.49E-08
ENSBTAG00000008629	*MTFR1*	14	31766665 ~ 31766665	1	4.63E-10
ENSBTAG00000011614	*PDE7A*	14	31820412 ~ 31851268	10	4.63E-10
ENSBTAG00000015229	*DNAJC5B*	14	32053374 ~ 32135874	6	9.43E-09
ENSBTAG00000021009	*TRIM55*	14	32186730 ~ 32186730	1	5.34E-10
ENSBTAG00000002192	*C8orf46*	14	32761431 ~ 32770549	5	3.95E-10
ENSBTAG00000044080	*CPA6*	14	33605805 ~ 33606681	2	3.5E-11
ENSBTAG00000022169	*PREX2*	14	34132990 ~ 34240707	20	4.82E-09
ENSBTAG00000022588	*C8orf34*	14	34435605 ~ 34442538	9	5.14E-10

*^*1*^Gene ID names were retrieved from Ensembl database using variant effect predictor (VEP) tools (McLaren et al., 2016) based on Bos taurus genome reference assembly UMD 3.1; ^*2*^Bos taurus autosome; ^*3*^Only first and last variant positions are presented for each gene; ^*4*^Represents the lowest P-value among the SNPs identified in a gene*.

In GWAS, only a few markers with the largest effects cross the significant threshold level through multiple hypothesis testing, and most variants fail to reach statistical significance, even though some of them are causal. To overcome the limitations of stringent criteria, we selected only the exonic SNPs (a total of 99,204) for further association study. The mixed linear model-based GWAS identified a total of 27 significant SNPs on BTA4, 6, and 14 (**Table 6** and Supplementary Figures S2, S3) for the CWT and EMA traits (*P* < 5.04 × 10^-7^). The significant exonic SNPs were harbored by 14 candidate genes, seven of which had already been detected when the SNPs in genic (exon and intron together) regions were used in the mixed linear model-based GWAS. Among the candidate genes, *TOX, COL1A2, PPARGC1A, PRKDC, IMPAD1, DNAJC5B*, and *CRH* were noteworthy (**Table 6**). Importantly, the coding variants on *COL1A2, PPARGC1A*, and *CRH* were significantly associated only with the exonic SNPs. The most significant SNP (rs110132121) was located in the 3′-UTR of *TOX* (*P* < 5.31 × 10^-15^) on BTA14 for CWT, followed by two synonymous SNPs (rs461493029 and rs449968016) in *PRKDC* (*P* < 6.22 × 10^-14^), also for CWT.

**Table 6:** Significant SNPs of exon regions in genome-wide association study (GWAS) for CWT and EMA traits in Korean Hanwoo cattle.

SNP	BTA	Position^1^ (bp)	Minor alleles	MAF^2^	*P*-value^3^	SNP location/effect^4^	Gene	Contri.^5^
Carcass weight								
Rs380188912	4	11079716	T	0.11	3.87E-07	3′ UTR	*GNG11*	0.012
Rs478237164	4	11661163	T	0.12	3.05E-08	Splice region	*COL1A2*	0.436
Rs133669403	6	44875315	A	0.04	1.19E-08	Missense	*PPARGC1A*	0.028
Rs208978122	6	44876187	A	0.04	1.19E-08	Synonymous	*PPARGC1A*	0.044
Rs381489766	6	46252102	A	0.14	1.06E-07	missense	*SEPSECS*	1.31E-05
Rs383916341	6	46255074	A	0.19	5.02E-07	Synonymous	*SEPSECS*	1.22E-04
Rs208065122	6	46464755	A	0.15	4.55E-10	Missense	*ZCCHC4*	0.106
Rs132745273	6	46492439	C	0.22	1.16E-08	Synonymous	*ANAPC4*	0.005
Rs109593072	14	13771715	A	0.13	1.25E-10	Synonymous	*MYC*	0.096
Rs110991194	14	13771721	C	0.17	8.55E-08	Synonymous	*MYC*	0.002
Rs461493029	14	21119128	G	0.10	6.22E-14	Synonymous	*PRKDC*	0.013
Rs449968016	14	21137279	T	0.09	6.22E-14	Missense	*PRKDC*	0.004
Rs381602905	14	25560744	A	0.19	7.54E-12	5′ UTR	*IMPAD1*	0.005
Rs41726594	14	26471148	T	0.22	2.96E-08	Synonymous	*NSMAF*	4.18E-06
Rs41726099	14	26479472	A	0.22	2.96E-08	Synonymous	*NSMAF*	4.41E-06
Rs41726103	14	26479946	C	0.22	2.96E-08	Synonymous	*NSMAF*	4.90E-06
Rs110132121	14	26631471	G	0.14	5.31E-15	3′ UTR	*TOX*	0.952
Rs207980725	14	29863346	A	0.10	7.53E-08	3′ UTR	*YTHDF3*	4.97E-07
Rs41734594	14	29863638	T	0.08	6.46E-10	3′ UTR	*YTHDF3*	2.45E-06
Rs109103375	14	32083468	T	0.11	9.22E-11	3′ UTR	*DNAJC5B*	3.66E-05
Rs109953090	14	32088652	C	0.11	9.22E-11	Missense	*DNAJC5B*	4.53E-05
Rs42682459	14	32164650	A	0.12	1.34E-09	Synonymous	*TRIM55*	1.03E-05
Rs109986397	14	32177663	A	0.11	3.42E-10	Synonymous	*TRIM55*	3.55E-05
Rs109714712	14	32213754	C	0.11	4.40E-11	Synonymous	*CRH*	1.30E-04
Rs381116984	14	32214109	T	0.11	4.40E-11	Missense	*CRH*	3.13E-05
Eye muscle area								
Rs461493029	14	21119128	G	0.10	4.82E-07	Synonymous	*PRKDC*	0.049
Rs449968016	14	21137279	T	0.10	4.82E-07	Missense	*PRKDC*	0.039

*^*1*^Positions are based on Bos taurus genome reference assembly UMD 3.1. ^*2*^minor allele frequency. ^*3*^Significant threshold at 5% level of genome-wide significance for Bonferroni correction was P = 5.04 × 10^-*7*^. ^*4*^Location of SNP variants or genes was performed as per cattle genome reference sequence (UMD 3.1) using SnpEff ver. 4.3p (Cingolani et al., 2012b) and variant effect predictor (VEP) tools (McLaren et al., 2016). ^*5*^Genetic contribution of each SNP was calculated using Bayesian mixture model*.

### Contributions of Genomic Variants

The SNP effects were estimated using BayesR to determine the proportion of genetic variance explained by individual SNPs and are presented in **Table 7** and Supplementary Figures S2–S5. We limited the analysis to the SNPs in the exon regions because of the heavy computational requirements of BayesR. The SNPs that had the largest effects for the investigated traits were located mostly on BTA2, 4, 6, 12, 14, 17, 19, and 24; however, these effects were small compared with the total genetic variance. Notably, 93–96% of the SNPs had close to zero effects, and the other 4–7% had different degrees of genetic contribution to the traits studied (**Table 6**). In particular, the proportion of SNPs that had the largest effects (10^-3^ × $\sigma_{A}^{2}$ and 10^-2^ × $\sigma_{A}^{2}$) varied between 0.26–0.41% of the total numbers but explained 33.42–62.73% of the total genetic variance.

**Table 7:** Estimates of number and proportion of exon region SNPs contributed in each mixture component by BayesR for carcass and meat quality traits in Korean Hanwoo cattle.

Trait	Nsnp	$\sigma_{G}^{2}$	Number of SNPs in mixture component			
			0 × $\sigma_{A}^{2}$	10^-4^ × $\sigma_{A}^{2}$	10^-3^ × $\sigma_{A}^{2}$	10^-2^ × $\sigma_{A}^{2}$
CWT	3979	580.93	95225	3652 (3.68)	292 (0.30)	35 (0.04)
			(95.99)	[215.72]	[169.74]	[194.65]
EMA	5001	21.97	94198	4638 (4.68)	346 (0.35)	22 (0.02)
			(94.95)	[10.45]	[7.57]	[3.92]
BFT	5305	4.17	93899	4899 (4.94)	390 (0.39)	16 (0.02)
			(94.65)	[2.07]	[1.64]	[0.46]
MS	6859	0.845	92345	6600 (6.66)	246 (0.25)	13 (0.01)
			(93.08)	[0.56]	[0.21]	[0.08]

*Nsnp, number of SNPs in model; $\sigma_{G}^{2}$, total genetic variance explained by the SNPs; values in parentheses are proportion of SNPs in each mixture component; $\sigma_{A}^{2}$, genetic variance explained by the respective mixture component and values are presented in the square brackets*. *CWT, carcass weight; EMA, eye muscle area; BFT, backfat thickness; MS, marbling score*.

## Discussion

Quantitative traits are controlled by the additive effects of a large number of genes spaced over an entire genome. Therefore, it is important to identify the genomic regions that contribute most to the genetic variations for complex traits like carcass and meat quality. In this study, we investigated for the first time, the genomic variances explained by different functional classes and performed GWAS using sequence level SNP information in Korean Hanwoo cattle.

### Partitioning of Genomic Variance by Chromosome

We found a linear but weak relationship between the variance explained by each chromosome and its length, which is consistent with the study of Jensen et al. (2012). They reported low *R^2^-*values (ranged between 0.11 and 0.21) for chromosomal variance on chromosomal lengths for complex traits in Holstein cattle. They also stated that aggregated chromosomal variance accounted for 96–97% of the total genomic variance, which is similar to our findings (Supplementary Table S2). Pimentel Eda et al. (2011) found that relatively broader linear relationships (*R^2^*) varied from 0.03 to 0.77 for milk production and milk composition traits in Holstein cattle, which is in partial agreement with the present study. Similar results were also found by Yang et al. (2011b) and Lee et al. (2012) who reported low to strong (*R^2^* = 0.03–0.80) linear relationships between genetic variance explained by each chromosome with its length for four complex traits and a complex genetic disorder, schizophrenia in human. Remarkably, we observed notable differences in genetic contribution among chromosomes of similar lengths, which is supported by the findings of Yang et al. (2011b). Taken together, these results indicate that the low *R^2^-*values between chromosomal lengths and their contributing genomic variances reflected only a weak relationship, which may be because genes that had large effects contributed a greater proportion of genomic variance for the harboring chromosome. The results of the present study also indicate that major genes or QTLs are not evenly segregated across the Hanwoo genome. For instance, *DGAT1* and *PLAG1* on BTA14 are known to make large contributions to genomic variance for carcass and milk traits in cattle, and accordingly we found the highest variance was attributed to BTA14, which is a small sized autosome. However, SNP density in the marker panel, statistical model used, types of traits investigated, and species of interest are major contributing factors to differences between our results and previous results. Overall, we found variable genomic contribution attributed across all chromosomes, which support a polygenic model for carcass and meat quality traits, and is similar to the findings of Pimentel Eda et al. (2011) and Jensen et al. (2012) for dairy traits in Holstein cattle.

### Partitioning of Genomic Variance by Functional Annotation and MAF Class

In agreement with our results, Abdollahi-Arpanahi et al. (2016) found that synonymous regions explained the largest proportion of genetic variance among six functional classes for body weight, hen-house egg production, and breast muscle measurement traits in broiler chicken. In human and cattle, Koufariotis et al. (2014) and Yang et al. (2011b) reported more genetic variances were attributed to genic regions than to intron and intergenic regions, which supports our findings. Moreover, the per SNP analysis revealed that both missense and synonymous classes had the largest contributions in total genetic variance (Koufariotis et al., 2014), which partially agrees with the present findings. Importantly, there has been increasing interest in synonymous SNPs, even though they do not change the amino acid in a polypeptide chain. Previous studies reported that synonymous mutations were associated with more than 50 human diseases (Sauna and Kimchi-Sarfaty, 2011), and also affected immature mRNA splicing, alteration of secondary structure of mRNA, stability of mRNA, protein folding, and the functions of translated proteins (Hunt et al., 2014). However, Morota et al. (2014) found that non-genic regions better explained genomic variance than genic regions for body weight and hen-house egg production traits in chicken, whereas for the breast muscle measurement trait, genic regions contributed more than non-genic regions. This variation with our findings might be due to differences in species of interest, number of SNPs investigated, and extent of LD between markers and QTLs. Overall, we found both genic and non-genic regions explained substantial amounts of genomic variances for the carcass and meat quality traits, which favors the infinitesimal theory and highlights the importance of SNPs spread over the entire genome.

van Binsbergen et al. (2015) reported that the frequency of low MAF increased proportionately with the advancement of SNP density and the proportions of low frequency alleles varied from 25 to 30% of the total SNPs in imputed sequence level SNP data and in whole-genome sequences. This result is in agreement with our present findings. Using sequence level SNP data in dairy cattle, Zhang et al. (2017) found the highest relative contribution in genomic variance was attributed to the common variants (MAF > 0.05–0.50) for production traits, whereas rare and low frequency alleles were more highly represents in the explained variance for fertility, longevity, and health-related traits. Their findings pointed toward a polygenic component of production traits and support our findings. Ogawa et al. (2016) reported a higher proportion of additive genetic variance was associated with common alleles where the MAF category ranged from 0.20 to 0.30 for the CWT trait in Japanese Black cattle. They also found that three major QTLs previously identified on BTA6, 8, and 14 were within the cited allele frequency range and potentially contributed to the higher genetic variance. However, the differences in MAF distribution for the CWT trait between previous and present findings may be associated primarily with SNP marker density. Taken together, these results suggest that common alleles make substantial contributions to the total genetic variance for quantitative traits and also support the present findings for carcass and meat quality traits in the Hanwoo population.

### GWAS and Contribution of Genomic Variants

Previous GWAS using both 50K and 777K data have revealed major QTL(s) on BTA14 associated with CWT and bovine stature in different cattle breeds including Hanwoo (Lee et al., 2013). Here, a wider range of significant SNPs was detected in BTA14 as well as in BTA4 and BTA6 using sequence level SNP information. These findings may help to identify more causal variants associated with economically important traits in cattle. Earlier studies reported genetic variants in and around *PLAG1* and a nearby major QTL on BTA14 for their associations with bovine stature (Karim et al., 2011), CWT (Nishimura et al., 2012), early life body weight, and peripubertal weight (Littlejohn et al., 2012), as well as birth weight (Utsunomiya et al., 2013) in different cattle populations. In our study, variants of neighboring genes of *PLAG1* were found to be significantly associated with CWT, but the most significant SNP marker (rs41724548) was located in *TOX*, which is 1.61 Mb distant from *PLAG1*, and also confirmed the previous findings of Lee et al. (2013). Based on 50K SNP chip data, Lee et al. (2013) reported that *PLAG1, CHCHD7, FAM110B, CYP7A1, SDCBP*, and *TOX* were positional and functional candidate genes for a CWT QTL in Hanwoo cattle, which supports our findings. In addition, they reported that the variants located near *PLAG1* and *CHCHD7* had non-significant associations with CWT, which is similar to the present findings. *TOX* acts as a transcription factor in the hypothalamus and plays a key role in the development of puberty in Brahman cattle (Fortes et al., 2012). Causal variants of *TOX* were associated with reproductive traits in Nellore cattle (de Camargo et al., 2015). Altogether, previous studies have reported that SNP variants associated with carcass traits were centered on *PLAG1*. However, we found SNP variants in an extended region between 20.7 and 34.4 Mb were associated with CWT, suggesting synergistic effects of multiple genes for the major QTL(s) on BTA14 in the Hanwoo population.

In previous studies, a QTL on BTA6 around the *NCAPG–LCORL* region was found to be associated with CWT and body frame size in Japanese Black cattle (Setoguchi et al., 2009, 2011) and birth, weaning, and yearling weight in crossbred beef cattle (Snelling et al., 2010). Setoguchi et al. (2011) found a LD block spanning a 591 kb region encompassed *FAM184B, DCAF16, NCAPG*, and *LCORL* where a causal variant (Ile442Met) was located in *NCAPG*. Recently, Xia et al. (2017) reported 11 significant SNPs associate with a skeleton trait in Simmental cattle that were located in or nearby *LAP3, FAM184B, LCORL*, and *NCAPG* on BTA6, which have been regarded as positional candidate regions for carcass and growth traits in cattle (Lindholm-Perry et al., 2011). Importantly, we found a number of significant markers within this region associated with CWT and EMA, and confirmed the previously reported association using sequence level SNP data for the first time in Hanwoo cattle.

In addition, similar to our study, a number of coding variants on *PPARGC1A, COL1A2*, and *CRH* have been documented for their association with growth, carcass, and meat quality traits in mammals including cattle. *PPARGC1A* encodes a transcriptional coactivator that regulates the genes involved in lipid and glucose metabolism, and has been regarded as a positional and functional candidate gene for carcass traits in beef cattle (Shin and Chung, 2013). The synonymous (c.396G > A) and missense (g.1181G > A) mutations of this gene had significant associations with body weight and average daily gain in Nanyang cattle (Li et al., 2014), as well as with growth, slaughter, and meat quality traits in Brangus steers (Soria et al., 2009). Besides, Shin and Chung (2013) reported two intronic SNPs in *PPARGC1A* to be significantly associated with the carcass trait EMA in Hanwoo, which supports our findings. *CRH* plays important roles for growth and development in mammals, and two coding SNPs (synonymous and missense) of this gene had significant association with CWT in our study. A missense mutation of *CRH* (G1084A) was significantly associated with the EMA trait in Hanwoo (Seong and Kong, 2015), which is in agreement with the present study. *COL1A2*, which encodes the pro-alpha2 chain of type I collagen, has been extensively investigated in human. Mutations in this gene were associated with several bone-related pathogenicity-like osteogenesis imperfecta and dental fluorosis. We found significant association with variants of *COL1A2* for CWT in Hanwoo. Above all, the coding variants detected in our study spanned three different genomic regions on BTA4, 6, and 14, whereas earlier studies documented major QTL(s) for carcass traits only on BTA14 in Hanwoo populations. Using sequence level SNP data, we detected two additional genomic regions (a 0.58 Mb region on BTA4 and a 1.61 Mb region on BTA6) in this study that may be new candidate loci for carcass traits in the investigated population. This information can be used to detect causal variants as well as in genomic selection programs in Hanwoo cattle.

Our results on the effect sizes of SNPs are in agreement with the infinitesimal theory as well as with the findings of Erbe et al. (2012) and Moser et al. (2015). Previous studies suggested that the minimum number of effective loci was between 400 and 4000 for capturing almost all genetic variances that accounted for milk production and disease resistance traits (Pimentel Eda et al., 2011; Erbe et al., 2012). In another investigation, Moser et al. (2015) reported that the number of SNPs with larger effects (10^-4^ × $\sigma_{A}^{2}$, 10^-3^ × $\sigma_{A}^{2}$, and 10^-2^ × $\sigma_{A}^{2}$) varied greatly (between 2633 and 9411) among seven human diseases. Moreover, they found that more than 96% of the SNPs were attributed with very small effects, close to zero. In our study, the number of large effect SNP variants in exon regions varied between 3979 (CWT) and 6859 (MS) among the investigated traits for explaining almost all of the total genetic variance, whereas the majority of the SNPs (>93%) were involved with the remaining genetic variance, which indicated the traits were polygenic in nature and were consistent with the previously reported findings in livestock and human. The types of traits investigated and the total number and category of SNP variants (exon, intron, or intergenic) included in the analysis might be major contributing factors for the differences between previous and present studies.

## Conclusion

Imputed genome sequence level data revealed the contributions of both genic and non-genic SNPs to phenotypic variations for four carcass and meat quality traits. Intragenic SNPs explained more genomic variance than intergenic variants, and the highest variance was attributed to synonymous SNPs. Genomic regions partitioned based on functional annotations, chromosome, and MAF category showed distinct differences in the variance explained for carcass and meat quality traits, and thus depicted different genetic architectures between the two types of traits. A wide range of significant SNPs and their contributions were established through this study. Some of these variants or genes that harbor them, first reported in this study, could be included in the genomic evaluation of quantitative traits in Hanwoo. Only 4–7% of the genic variants potentially contributed to the total explained genetic variance, while the remaining thousands had close to zero contribution and largely point toward the polygenic composition of these traits.

## Data Accessibility

The high density SNP genotypic data and full genome sequence data of Korean Hanwoo cattle used in this study are deposited and available at digital repository of NIAS, South Korea (website) and would be available to the interested researcher upon the request.

## Author Contributions

SeL and MB conceived and designed the study. MB drafted the manuscript. DL and CG were responsible for imputation of 50K and 777K genotype data to sequence level. DL and SoL were responsible for phenotypic data collection. SoL and YK contributed in quality control of genotype data, partitioning of genome and SNP annotation. MB and YK performed GWAS. All authors read and agreed on the contents of manuscript.

## Conflict of Interest Statement

The authors declare that the research was conducted in the absence of any commercial or financial relationships that could be construed as a potential conflict of interest.

## Footnotes

1 https://github.com/syntheke/bayesR
