# Supplementary material for: Functional Partitioning of Genomic Variance and Genome-Wide Association Study for Carcass Traits in Korean Hanwoo Cattle Using Imputed Sequence Level SNP Data
Source: Front Genet. 2018 Jun 22;9:217. doi: 10.3389/fgene.2018.00217 (PMC6024024; doi:10.3389/fgene.2018.00217)
Supplement: Supplementary file 1 [file Table_1.DOCX]

**Supplementary Material**

**Partitioning of Genomic Variance and Genome-wide Association Study for Quantitative Traits in Korean Hanwoo Cattle Using Imputed Sequence Level SNP Data**

**Mohammad Shamsul Alam Bhuiyan^1,5,*^, Dajeong Lim^2,*^, Mina Park^3,*^, Soo Hyun Lee^1^, Yeong Kuk Kim^1^, Cedric Gondro^4^ , Byeong Ho Park^3,$^ and Seung Hwan Lee^1,$^**

**^*^Correspondence:** Seung Hwan Lee, PhD; [slee46@cnu.ac.kr](mailto:slee46@cnu.ac.kr);

**Byeong Ho Park, Ph.D.** [bhpark70@korea.kr](mailto:bhpark70@korea.kr)

**TABLE S1 |** Descriptive statistics for carcass and meat quality traits in Korean Hanwoo cattle

| **Trait^1^** | **Mean** | **SD** | **Min** | **Max** | **CV** |
| --- | --- | --- | --- | --- | --- |
| CW (kg) | 361.20 | 40.31 | 160 | 518 | 11.16 |
| EMA (cm^2^) | 81.72 | 8.93 | 41 | 123 | 10.92 |
| BFT (mm) | 8.57 | 3.52 | 1 | 28 | 41.13 |
| MS (1-9 scale) | 3.23 | 1.49 | 1 | 9 | 46.30 |

^1^See Table 2 for trait abbreviations.

**TABLE S2 |** Estimates of variance explained by 29 autosomes for four carcass and meat quality traits in Korean Hanwoo cattle

| **Chr.** | **Length (Mb)** | **CWT** | | **EMA** | | **BFT** | | **MS** | |
| --- | --- | --- | --- | --- | --- | --- | --- | --- | --- |
|  |  | $\boldsymbol{h}^{\boldsymbol{2}}$ | **SE** | $\boldsymbol{h}^{\boldsymbol{2}}$ | **SE** | $\boldsymbol{h}^{\boldsymbol{2}}$ | **SE** | $\boldsymbol{h}^{\boldsymbol{2}}$ | **SE** |
| 1 | 158.3371 | 0.0203 | 0.0160 | 0.0158 | 0.0169 | 0.0010 | 0.0132 | 0.0232 | 0.0178 |
| 2 | 137.0604 | 0.0296 | 0.0178 | 0.0271 | 0.0181 | 0.0207 | 0.0163 | 0.0268 | 0.0180 |
| 3 | 121.4304 | 0.0317 | 0.0181 | 0.0357 | 0.0177 | 0.0055 | 0.0122 | 0.0446 | 0.0198 |
| 4 | 120.8297 | 0.0702 | 0.0202 | 0.0141 | 0.0159 | 0.0442 | 0.0197 | 0.0283 | 0.0183 |
| 5 | 121.1914 | 0.0085 | 0.0133 | 0.0094 | 0.0142 | 0.0315 | 0.0171 | 0.0472 | 0.0197 |
| 6 | 119.4587 | 0.0656 | 0.0194 | 0.0646 | 0.0212 | 0.0000 | 0.0126 | 0.0000 | 0.0123 |
| 7 | 112.6397 | 0.0150 | 0.0145 | 0.0000 | 0.0139 | 0.0275 | 0.0165 | 0.0034 | 0.0130 |
| 8 | 113.3848 | 0.0122 | 0.0127 | 0.0123 | 0.0139 | 0.0000 | 0.0141 | 0.0221 | 0.0156 |
| 9 | 105.7083 | 0.0202 | 0.0145 | 0.0152 | 0.0146 | 0.0237 | 0.0160 | 0.0038 | 0.0125 |
| 10 | 104.3050 | 0.0208 | 0.0162 | 0.0207 | 0.0154 | 0.0220 | 0.0169 | 0.0450 | 0.0206 |
| 11 | 107.3108 | 0.0160 | 0.0131 | 0.0145 | 0.0146 | 0.0351 | 0.0169 | 0.0110 | 0.0139 |
| 12 | 91.1631 | 0.0053 | 0.0100 | 0.0113 | 0.0130 | 0.0186 | 0.0152 | 0.0003 | 0.0115 |
| 13 | 84.2404 | 0.0000 | 0.0107 | 0.0076 | 0.0125 | 0.0316 | 0.0167 | 0.0071 | 0.0133 |
| 14 | 84.6484 | 0.0890 | 0.0205 | 0.0435 | 0.0169 | 0.0000 | 0.0121 | 0.0130 | 0.0142 |
| 15 | 85.2967 | 0.0115 | 0.0120 | 0.0000 | 0.0112 | 0.0196 | 0.0139 | 0.0000 | 0.0129 |
| 16 | 81.7247 | 0.0126 | 0.0134 | 0.0096 | 0.0144 | 0.0265 | 0.0156 | 0.0000 | 0.0128 |
| 17 | 75.1586 | 0.0000 | 0.0121 | 0.0105 | 0.0133 | 0.0000 | 0.0140 | 0.0253 | 0.0161 |
| 18 | 66.0040 | 0.0068 | 0.0123 | 0.0136 | 0.0133 | 0.0101 | 0.0139 | 0.0323 | 0.0153 |
| 19 | 64.0575 | 0.0432 | 0.0182 | 0.0429 | 0.0178 | 0.0220 | 0.0155 | 0.0422 | 0.0173 |
| 20 | 72.0427 | 0.0049 | 0.0115 | 0.0066 | 0.0117 | 0.0056 | 0.0117 | 0.0200 | 0.0147 |
| 21 | 71.5991 | 0.0196 | 0.0139 | 0.0000 | 0.0104 | 0.0033 | 0.0124 | 0.0000 | 0.0131 |
| 22 | 61.4359 | 0.0282 | 0.0143 | 0.0000 | 0.0117 | 0.0207 | 0.0138 | 0.0192 | 0.0123 |
| 23 | 52.5301 | 0.0089 | 0.0109 | 0.0010 | 0.0102 | 0.0233 | 0.0155 | 0.0231 | 0.0152 |
| 24 | 62.7149 | 0.0000 | 0.0103 | 0.0055 | 0.0118 | 0.0106 | 0.0122 | 0.0000 | 0.0106 |
| 25 | 42.9042 | 0.0066 | 0.0082 | 0.0066 | 0.0095 | 0.0174 | 0.0135 | 0.0055 | 0.0098 |
| 26 | 51.6815 | 0.0000 | 0.0115 | 0.0000 | 0.0112 | 0.0023 | 0.0095 | 0.0230 | 0.0142 |
| 27 | 45.4079 | 0.0070 | 0.0099 | 0.0125 | 0.0118 | 0.0000 | 0.0099 | 0.0000 | 0.0099 |
| 28 | 46.3125 | 0.0000 | 0.0076 | 0.0056 | 0.0099 | 0.0000 | 0.0107 | 0.0090 | 0.0109 |
| 29 | 51.5052 | 0.0000 | 0.0086 | 0.0161 | 0.0124 | 0.0060 | 0.0097 | 0.0242 | 0.0146 |
| **Total** | 2512.0835 | 0.5533 | 0.0482 | 0.4224 | 0.0481 | 0.4285 | 0.0486 | 0.4999 | 0.0479 |

^1^See Table 2 for trait abbreviations.

**TABLE S3 |** Significant SNPs of genic regions associated with CWT and EMA traits in Korean Hanwoo cattle

| **SNP marker** | **BTA** | **Position^1^ (bp)** | **Minor Alleles** | **MAF^2^** | **P-value^3^** | **SNP location/effect^4^** | **Gene** |
| --- | --- | --- | --- | --- | --- | --- | --- |
| rs476493553 | 6 | 38577764 | T | 0.052 | 1.22E-08 | intron | *LAP3* |
| 6:38580679 | 6 | 38580679 | G | 0.037 | 9E-12 | intron | *LAP3* |
| rs432843769 | 6 | 38581067 | A | 0.052 | 9.9E-09 | intron | *LAP3* |
| rs468565692 | 6 | 38581337 | C | 0.052 | 9.9E-09 | intron | *LAP3* |
| rs451798201 | 6 | 38581510 | G | 0.052 | 9.9E-09 | intron | *LAP3* |
| rs454017544 | 6 | 38583582 | A | 0.052 | 9.9E-09 | intron | *LAP3* |
| rs109438687^a^ | 6 | 38648218 | C | 0.053 | 2.47E-17 | intron | *FAM184B* |
| rs109467519^a^ | 6 | 38648565 | T | 0.053 | 2.47E-17 | intron | *FAM184B* |
| rs109355965^a^ | 6 | 38657124 | C | 0.052 | 1.06E-16 | intron | *FAM184B* |
| rs384833298^a^ | 6 | 38657286 | A | 0.052 | 1.06E-16 | intron | *FAM184B* |
| rs110766531^a^ | 6 | 38670165 | A | 0.052 | 1.06E-16 | intron | *FAM184B* |
| rs208757496 | 6 | 38794618 | A | 0.078 | 1.35E-08 | intron | *NCAPG* |
| rs109240064 | 6 | 38795382 | C | 0.078 | 1.35E-08 | intron | *NCAPG* |
| rs109647759 | 6 | 38795511 | C | 0.078 | 1.35E-08 | intron | *NCAPG* |
| rs110628070 | 6 | 38798040 | T | 0.078 | 1.35E-08 | intron | *NCAPG* |
| rs109998054 | 6 | 38804348 | A | 0.078 | 1.35E-08 | intron | *NCAPG* |
| rs108945859 | 6 | 38849296 | C | 0.078 | 1.35E-08 | intron | *LCORL* |
| rs110305942 | 6 | 38852093 | G | 0.078 | 1.35E-08 | intron | *LCORL* |
| rs110891752 | 6 | 38852378 | G | 0.067 | 2.04E-11 | intron | *LCORL* |
| rs109713296 | 6 | 38858177 | C | 0.078 | 1.35E-08 | intron | *LCORL* |
| rs385185550 | 6 | 38865025 | C | 0.065 | 2.16E-12 | intron | *LCORL* |
| rs109711776 | 6 | 38871462 | T | 0.078 | 1.35E-08 | intron | *LCORL* |
| rs109732906 | 6 | 38872172 | A | 0.065 | 2.16E-12 | intron | *LCORL* |
| rs111024484 | 6 | 38872917 | T | 0.078 | 1.35E-08 | intron | *LCORL* |
| rs207644359 | 6 | 38875198 | C | 0.078 | 1.35E-08 | intron | *LCORL* |
| rs208932260 | 6 | 38883815 | G | 0.078 | 1.35E-08 | intron | *LCORL* |
| rs110658091 | 6 | 38900113 | C | 0.078 | 1.35E-08 | intron | *LCORL* |
| 6:41262050 | 6 | 41262050 | A | 0.039 | 5.4E-10 | intron | *SLIT2* |
| 6:41284760 | 6 | 41284760 | T | 0.039 | 5.4E-10 | intron | *SLIT2* |
| rs470479052^a^ | 6 | 41292118 | A | 0.047 | 6.06E-12 | intron | *SLIT2* |
| 6:41342263 | 6 | 41342263 | A | 0.039 | 5.4E-10 | intron | *SLIT2* |
| rs437831306 | 6 | 41409518 | C | 0.041 | 1.02E-08 | intron | *SLIT2* |
| 6:41415218 | 6 | 41415218 | T | 0.040 | 3.34E-09 | intron | *SLIT2* |
| rs384017132 | 6 | 41438938 | G | 0.039 | 1.23E-09 | intron | *SLIT2* |
| 6:41439005 | 6 | 41439005 | C | 0.039 | 1.23E-09 | intron | *SLIT2* |
| 6:41463763 | 6 | 41463763 | A | 0.039 | 1.23E-09 | intron | *SLIT2* |
| 6:41526051 | 6 | 41526051 | C | 0.039 | 1.23E-09 | intron | *SLIT2* |
| 6:41845249 | 6 | 41845249 | C | 0.038 | 2.84E-09 | intron | *KCNIP4* |
| rs471325385 | 6 | 41900486 | A | 0.038 | 2.84E-09 | intron | *KCNIP4* |
| rs385384411 | 14 | 20603742 | T | 0.096 | 4.46E-12 | intron | *-* |
| rs434541169 | 14 | 20753321 | T | 0.096 | 5.37E-11 | intron | *SPIDR* |
| rs382735458 | 14 | 20843282 | A | 0.161 | 5.95E-09 | intron | *SPIDR* |
| rs449665799 | 14 | 20854191 | A | 0.120 | 1.09E-10 | intron | *SPIDR* |
| rs467964819 | 14 | 20856649 | T | 0.120 | 1.09E-10 | intron | *SPIDR* |
| rs442683137 | 14 | 20875591 | T | 0.120 | 1.09E-10 | intron | *SPIDR* |
| rs478021634 | 14 | 21043161 | G | 0.097 | 6.7E-13 | intron | *PRKDC* |
| rs480288289 | 14 | 21043434 | A | 0.097 | 6.7E-13 | intron | *PRKDC* |
| rs436815880 | 14 | 21052447 | A | 0.097 | 6.7E-13 | intron | *PRKDC* |
| rs468797213 | 14 | 21055569 | T | 0.097 | 6.7E-13 | intron | *PRKDC* |
| rs380576559 | 14 | 21062075 | T | 0.097 | 6.7E-13 | intron | *PRKDC* |
| rs378042435 | 14 | 21062747 | A | 0.097 | 6.7E-13 | intron | *PRKDC* |
| rs384216424 | 14 | 21064313 | A | 0.097 | 6.7E-13 | intron | *PRKDC* |
| rs461363876 | 14 | 21067479 | A | 0.097 | 6.7E-13 | intron | *PRKDC* |
| rs480738237 | 14 | 21075396 | G | 0.097 | 6.7E-13 | intron | *PRKDC* |
| rs380835402 | 14 | 21075618 | A | 0.121 | 1.15E-12 | intron | *PRKDC* |
| rs476988183 | 14 | 21080976 | T | 0.120 | 9.02E-13 | intron | *PRKDC* |
| rs381180236 | 14 | 21081948 | A | 0.120 | 9.02E-13 | intron | *PRKDC* |
| rs377944775 | 14 | 21084174 | A | 0.120 | 9.02E-13 | intron | *PRKDC* |
| rs211246384 | 14 | 21091883 | C | 0.097 | 6.7E-13 | intron | *PRKDC* |
| rs41624087 | 14 | 21094537 | A | 0.097 | 6.7E-13 | intron | *PRKDC* |
| rs480824921 | 14 | 21100653 | C | 0.097 | 6.7E-13 | intron | *PRKDC* |
| rs384646179 | 14 | 21111076 | C | 0.097 | 6.7E-13 | intron | *PRKDC* |
| rs445357152 | 14 | 21113212 | A | 0.097 | 6.7E-13 | intron | *PRKDC* |
| rs472138483 | 14 | 21114256 | A | 0.097 | 6.7E-13 | intron | *PRKDC* |
| rs436411613 | 14 | 21115613 | A | 0.097 | 6.7E-13 | intron | *PRKDC* |
| rs461493029 | 14 | 21119128 | G | 0.097 | 6.7E-13 | synonymous | *PRKDC* |
| rs41581313 | 14 | 21123207 | T | 0.097 | 6.7E-13 | intron | *PRKDC* |
| rs379681330 | 14 | 21123872 | T | 0.097 | 6.7E-13 | intron | *PRKDC* |
| rs382898927 | 14 | 21126095 | A | 0.097 | 6.7E-13 | intron | *PRKDC* |
| rs379370583 | 14 | 21129250 | A | 0.097 | 6.7E-13 | intron | *PRKDC* |
| rs380015967 | 14 | 21130523 | A | 0.097 | 6.7E-13 | intron | *PRKDC* |
| rs385298992 | 14 | 21130698 | T | 0.097 | 6.7E-13 | intron | *PRKDC* |
| rs449968016 | 14 | 21137279 | T | 0.097 | 6.7E-13 | missense | *PRKDC* |
| rs378138310 | 14 | 21144233 | T | 0.097 | 6.7E-13 | intron | *PRKDC* |
| rs384540527 | 14 | 21144432 | A | 0.097 | 6.7E-13 | intron | *PRKDC* |
| rs382372593 | 14 | 21147869 | T | 0.097 | 6.7E-13 | intron | *PRKDC* |
| rs382834202 | 14 | 21151156 | T | 0.097 | 6.7E-13 | intron | *PRKDC* |
| rs211204541 | 14 | 24332803 | G | 0.098 | 1.73E-16 | intron | *XKR4* |
| rs210090325 | 14 | 24343693 | A | 0.098 | 1.73E-16 | intron | *XKR4* |
| rs42649781 | 14 | 24462537 | A | 0.240 | 4.58E-09 | intron | *XKR4* |
| rs42649780 | 14 | 24466047 | A | 0.240 | 4.58E-09 | intron | *XKR4* |
| rs42650124 | 14 | 24470335 | T | 0.242 | 1.36E-08 | intron | *XKR4* |
| rs110543321 | 14 | 24471148 | C | 0.242 | 1.36E-08 | intron | *XKR4* |
| rs136622238 | 14 | 24471474 | C | 0.242 | 1.36E-08 | intron | *XKR4* |
| rs209572821 | 14 | 24474674 | C | 0.242 | 1.36E-08 | intron | *XKR4* |
| rs381796151 | 14 | 25546508 | T | 0.193 | 9.27E-12 | intron | *IMPAD1* |
| rs382587753 | 14 | 25558057 | A | 0.189 | 1.29E-11 | intron | *IMPAD1* |
| rs385158238 | 14 | 25558093 | T | 0.189 | 1.29E-11 | intron | *IMPAD1* |
| rs380434832 | 14 | 25558645 | G | 0.189 | 1.29E-11 | intron | *IMPAD1* |
| rs378339662 | 14 | 25559958 | T | 0.189 | 1.29E-11 | intron | *IMPAD1* |
| rs381602905 | 14 | 25560744 | A | 0.189 | 1.29E-11 | 5' UTR | *IMPAD1* |
| rs41614868 | 14 | 26351959 | C | 0.124 | 2.82E-15 | intron | *CYP7A1* |
| rs209439851 | 14 | 26443481 | A | 0.128 | 5.75E-15 | intron | *SDCBP* |
| rs41720539 | 14 | 26445603 | A | 0.128 | 5.75E-15 | intron | *SDCBP* |
| rs41726566 | 14 | 26496858 | A | 0.125 | 3.62E-16 | intron | *NSMAF* |
| rs110132121 | 14 | 26631471 | G | 0.142 | 3.56E-12 | 3' UTR | *TOX* |
| rs110635436 | 14 | 26634085 | T | 0.142 | 3.56E-12 | intron | *TOX* |
| rs111028490 | 14 | 26636169 | G | 0.143 | 2.39E-12 | intron | *TOX* |
| rs110790018 | 14 | 26636257 | A | 0.143 | 2.39E-12 | intron | *TOX* |
| rs109938685 | 14 | 26637969 | A | 0.142 | 3.56E-12 | intron | *TOX* |
| rs136316119 | 14 | 26639406 | T | 0.142 | 2.09E-12 | intron | *TOX* |
| rs109748853 | 14 | 26641886 | T | 0.142 | 2.09E-12 | intron | *TOX* |
| rs41725174 | 14 | 26642947 | G | 0.128 | 1.01E-14 | intron | *TOX* |
| rs136253179 | 14 | 26644261 | T | 0.142 | 2.09E-12 | intron | *TOX* |
| rs110460774 | 14 | 26645529 | C | 0.142 | 2.09E-12 | intron | *TOX* |
| rs109347001 | 14 | 26645820 | A | 0.142 | 2.09E-12 | intron | *TOX* |
| rs385571882 | 14 | 26646557 | G | 0.142 | 2.09E-12 | intron | *TOX* |
| rs110560164 | 14 | 26648246 | C | 0.142 | 2.09E-12 | intron | *TOX* |
| rs136725588 | 14 | 26650009 | G | 0.150 | 3.82E-09 | intron | *TOX* |
| rs109931944 | 14 | 26650392 | A | 0.147 | 8.39E-10 | intron | *TOX* |
| rs109073664 | 14 | 26651141 | T | 0.126 | 7.39E-16 | intron | *TOX* |
| rs110032645 | 14 | 26655194 | A | 0.144 | 2.45E-10 | intron | *TOX* |
| rs109374728 | 14 | 26656398 | A | 0.122 | 5.57E-17 | intron | *TOX* |
| rs137238508 | 14 | 26658329 | T | 0.144 | 2.45E-10 | intron | *TOX* |
| rs111021769 | 14 | 26662633 | A | 0.144 | 2.45E-10 | intron | *TOX* |
| rs109258546 | 14 | 26665453 | C | 0.144 | 2.45E-10 | intron | *TOX* |
| rs109560657 | 14 | 26666892 | T | 0.144 | 4.03E-10 | intron | *TOX* |
| rs135927531 | 14 | 26669442 | T | 0.144 | 4.03E-10 | intron | *TOX* |
| rs41724619 | 14 | 26686733 | A | 0.122 | 1.04E-16 | intron | *TOX* |
| rs41724552 | 14 | 26738155 | C | 0.171 | 2.22E-09 | intron | *TOX* |
| rs41724548 | 14 | 26743126 | C | 0.119 | 3.16E-17 | intron | *TOX* |
| rs41724547 | 14 | 26746062 | C | 0.119 | 3.16E-17 | intron | *TOX* |
| rs41724546 | 14 | 26749008 | G | 0.119 | 3.16E-17 | intron | *TOX* |
| rs41724541 | 14 | 26764020 | T | 0.121 | 1.94E-15 | intron | *TOX* |
| rs41724540 | 14 | 26764679 | T | 0.151 | 1.19E-11 | intron | *TOX* |
| rs41724537 | 14 | 26765679 | G | 0.138 | 1.86E-12 | intron | *TOX* |
| rs41724536 | 14 | 26766010 | T | 0.121 | 1.94E-15 | intron | *TOX* |
| rs41724535 | 14 | 26768630 | T | 0.151 | 1.19E-11 | intron | *TOX* |
| rs110320866 | 14 | 26773332 | A | 0.151 | 1.19E-11 | intron | *TOX* |
| rs41724030 | 14 | 26773726 | T | 0.151 | 1.19E-11 | intron | *TOX* |
| rs108988945 | 14 | 26775729 | T | 0.151 | 1.19E-11 | intron | *TOX* |
| rs41724028 | 14 | 26776546 | G | 0.137 | 1.28E-12 | intron | *TOX* |
| rs41724018 | 14 | 26786648 | T | 0.137 | 1.28E-12 | intron | *TOX* |
| rs41724019 | 14 | 26787240 | A | 0.155 | 9.37E-11 | intron | *TOX* |
| rs41724020 | 14 | 26787374 | A | 0.155 | 9.37E-11 | intron | *TOX* |
| rs41724015 | 14 | 26794576 | C | 0.137 | 1.28E-12 | intron | *TOX* |
| rs41627959 | 14 | 26804892 | C | 0.137 | 1.28E-12 | intron | *TOX* |
| rs41579374 | 14 | 26820702 | A | 0.156 | 6.18E-10 | intron | *TOX* |
| rs210783684 | 14 | 26827584 | C | 0.165 | 2.36E-10 | intron | *TOX* |
| rs42406068 | 14 | 26828519 | T | 0.165 | 2.36E-10 | intron | *TOX* |
| rs42406058 | 14 | 26848418 | G | 0.119 | 3.16E-17 | intron | *TOX* |
| rs42406039 | 14 | 26859737 | T | 0.119 | 3.16E-17 | intron | *TOX* |
| rs42404967 | 14 | 26941314 | G | 0.164 | 1.25E-08 | intron | *TOX* |
| rs380215837 | 14 | 28702223 | A | 0.116 | 1.88E-09 | intron | *ASPH* |
| rs110103595 | 14 | 28705829 | A | 0.120 | 1.09E-08 | intron | *ASPH* |
| rs136400120 | 14 | 28706578 | G | 0.120 | 1.09E-08 | intron | *ASPH* |
| rs136506581 | 14 | 28708569 | A | 0.120 | 1.09E-08 | intron | *ASPH* |
| rs207552329 | 14 | 28709315 | T | 0.116 | 1.88E-09 | intron | *ASPH* |
| rs379672546 | 14 | 28712341 | A | 0.116 | 1.88E-09 | intron | *ASPH* |
| rs110883161 | 14 | 31058827 | C | 0.174 | 1.49E-08 | intron | *CYP7B1* |
| rs209689999 | 14 | 31059125 | C | 0.174 | 1.49E-08 | intron | *CYP7B1* |
| rs109978429 | 14 | 31064078 | A | 0.174 | 1.49E-08 | intron | *CYP7B1* |
| rs110858505 | 14 | 31766665 | A | 0.092 | 4.63E-10 | intron | *MTFR1* |
| rs110141788 | 14 | 31820412 | A | 0.092 | 4.63E-10 | intron | *PDE7A* |
| rs136349024 | 14 | 31820574 | T | 0.092 | 4.63E-10 | intron | *PDE7A* |
| rs110629119 | 14 | 31823139 | A | 0.092 | 4.63E-10 | intron | *PDE7A* |
| rs109714322 | 14 | 31824400 | G | 0.092 | 4.63E-10 | intron | *PDE7A* |
| rs110901383 | 14 | 31826178 | A | 0.092 | 4.63E-10 | intron | *PDE7A* |
| rs109382700 | 14 | 31828122 | C | 0.092 | 4.63E-10 | intron | *PDE7A* |
| rs110522740 | 14 | 31833227 | T | 0.092 | 4.63E-10 | intron | *PDE7A* |
| rs110044282 | 14 | 31843402 | T | 0.092 | 4.63E-10 | intron | *PDE7A* |
| rs110110600 | 14 | 31844190 | A | 0.092 | 4.63E-10 | intron | *PDE7A* |
| rs109864374 | 14 | 31851268 | C | 0.092 | 4.63E-10 | intron | *PDE7A* |
| rs210399433 | 14 | 32053374 | C | 0.116 | 9.43E-09 | intron | *DNAJC5B* |
| rs208101544 | 14 | 32077100 | C | 0.093 | 6.77E-11 | intron | *DNAJC5B* |
| rs209232609 | 14 | 32117083 | A | 0.093 | 8.47E-11 | intron | *DNAJC5B* |
| rs208970307 | 14 | 32126069 | C | 0.093 | 8.47E-11 | intron | *DNAJC5B* |
| rs385467852 | 14 | 32130656 | C | 0.098 | 4.22E-10 | intron | *DNAJC5B* |
| rs384145098 | 14 | 32135874 | C | 0.093 | 8.47E-11 | intron | *DNAJC5B* |
| 14:32186730 | 14 | 32186730 | C | 0.094 | 5.34E-10 | intron | *TRIM55* |
| rs41726844 | 14 | 32761431 | A | 0.122 | 1.29E-10 | intron | *C8orf46* |
| rs433335392 | 14 | 32761472 | A | 0.095 | 3.95E-10 | intron | *C8orf46* |
| rs378271268 | 14 | 32762165 | C | 0.095 | 3.95E-10 | intron | *C8orf46* |
| rs207963457 | 14 | 32763433 | A | 0.122 | 1.29E-10 | intron | *C8orf46* |
| rs41726852 | 14 | 32770549 | T | 0.114 | 2.2E-11 | intron | *C8orf46* |
| rs210623229 | 14 | 33605805 | T | 0.093 | 3.5E-11 | intron | *CPA6* |
| 14:33606681 | 14 | 33606681 | A | 0.093 | 3.5E-11 | intron | *CPA6* |
| rs207828115 | 14 | 34132990 | A | 0.112 | 2.05E-12 | intron | *PREX2* |
| rs209018155 | 14 | 34139194 | T | 0.113 | 8E-13 | intron | *PREX2* |
| rs211042876 | 14 | 34144633 | A | 0.113 | 8E-13 | intron | *PREX2* |
| rs109169231 | 14 | 34148235 | A | 0.113 | 8E-13 | intron | *PREX2* |
| rs208529032 | 14 | 34150502 | T | 0.113 | 8E-13 | intron | *PREX2* |
| rs207547654 | 14 | 34157781 | G | 0.113 | 8E-13 | intron | *PREX2* |
| rs379734181 | 14 | 34161914 | T | 0.113 | 8E-13 | intron | *PREX2* |
| rs209286213 | 14 | 34183059 | T | 0.113 | 8E-13 | intron | *PREX2* |
| rs210743676 | 14 | 34189984 | G | 0.113 | 8E-13 | intron | *PREX2* |
| rs379507012 | 14 | 34193453 | T | 0.113 | 8E-13 | intron | *PREX2* |
| rs137454864 | 14 | 34194915 | C | 0.113 | 3.13E-13 | intron | *PREX2* |
| rs207855612 | 14 | 34198652 | A | 0.113 | 3.13E-13 | intron | *PREX2* |
| rs210640333 | 14 | 34216413 | T | 0.115 | 9.09E-14 | intron | *PREX2* |
| rs109737298 | 14 | 34219324 | A | 0.115 | 9.09E-14 | intron | *PREX2* |
| rs110204308 | 14 | 34219758 | A | 0.115 | 9.09E-14 | intron | *PREX2* |
| rs134840045 | 14 | 34223518 | A | 0.127 | 4.82E-09 | intron | *PREX2* |
| rs210191458 | 14 | 34223684 | G | 0.116 | 7.95E-14 | intron | *PREX2* |
| rs211030164 | 14 | 34233185 | T | 0.115 | 9.09E-14 | intron | *PREX2* |
| rs207528588 | 14 | 34238097 | C | 0.115 | 9.09E-14 | intron | *PREX2* |
| rs208809071 | 14 | 34240707 | T | 0.114 | 1.04E-13 | intron | *PREX2* |
| rs209167562 | 14 | 34435605 | G | 0.124 | 5.14E-10 | intron | *C8orf34* |
| rs133072256 | 14 | 34435666 | T | 0.124 | 5.14E-10 | intron | *C8orf34* |
| rs110532704 | 14 | 34436069 | C | 0.124 | 5.14E-10 | intron | *C8orf34* |
| rs210453272 | 14 | 34437265 | T | 0.124 | 5.14E-10 | intron | *C8orf34* |
| rs207742579 | 14 | 34437640 | A | 0.124 | 5.14E-10 | intron | *C8orf34* |
| rs210948979 | 14 | 34437670 | T | 0.124 | 5.14E-10 | intron | *C8orf34* |
| rs109677034 | 14 | 34438747 | A | 0.124 | 5.14E-10 | intron | *C8orf34* |
| rs211414174 | 14 | 34441381 | A | 0.124 | 5.14E-10 | intron | *C8orf34* |
| rs211672010 | 14 | 34442538 | C | 0.124 | 5.14E-10 | intron | *C8orf34* |

^1^SNP positions are based on *Bos taurus* genome reference assembly UMD 3.1, ^2^minor allele frequency, ^3^signiﬁcant threshold at 5% level of genome-wide signiﬁcance for Bonferroni correction was P = 1.49 × 10^-8^

^4^locations of SNP variants or genes were identified as per cattle genome reference sequence (UMD 3.1) using SnpEff ver. 4.3p (Cingolani et al., 2012) and Variant Effect Predictor (VEP) tools (McLaren et al., 2016),

^a^denotes significant association both for CWT and EMA traits.


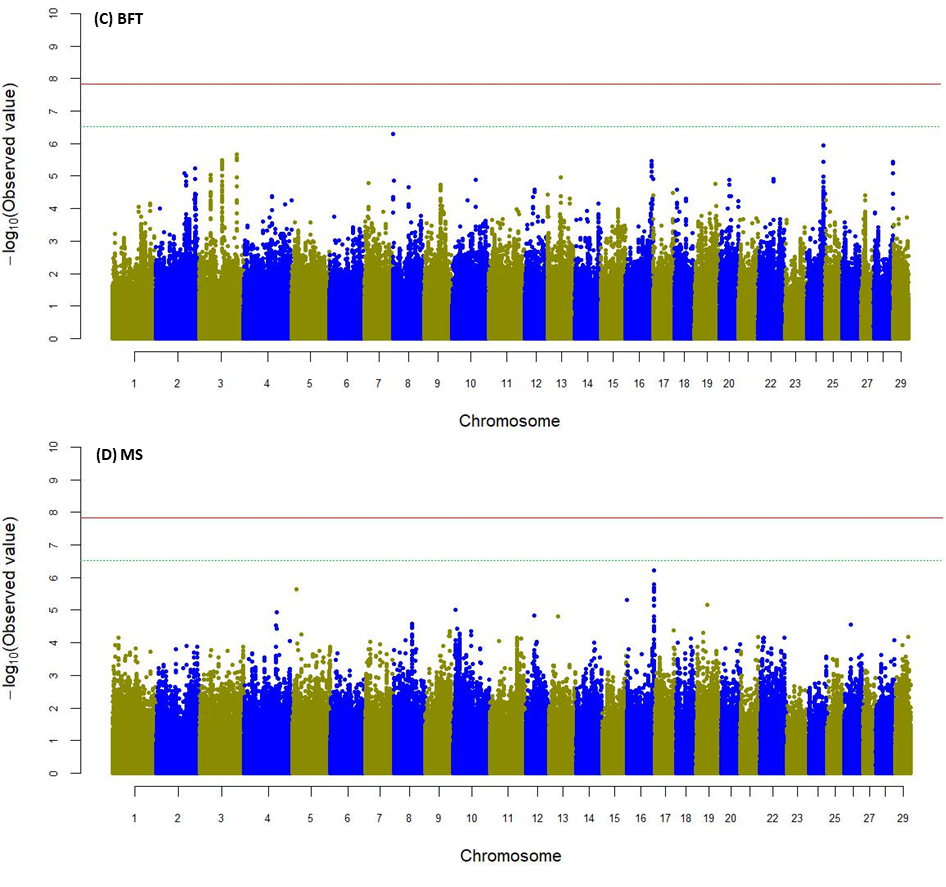


**FIGURE S1 |** Manhattan plot of GWAS using SNPs of genic (exon and intron) regions for BFT (C) and MS (D) traits where Y-axis defines -log10 (P) value against their respective positions on each chromosome (X-axis). The horizontal solid and dot lines indicate the Bonferroni adjusted significant (P<1.49×10^-8^) and suggestive (P <2.99×10^-7^) thresholds level, respectively.


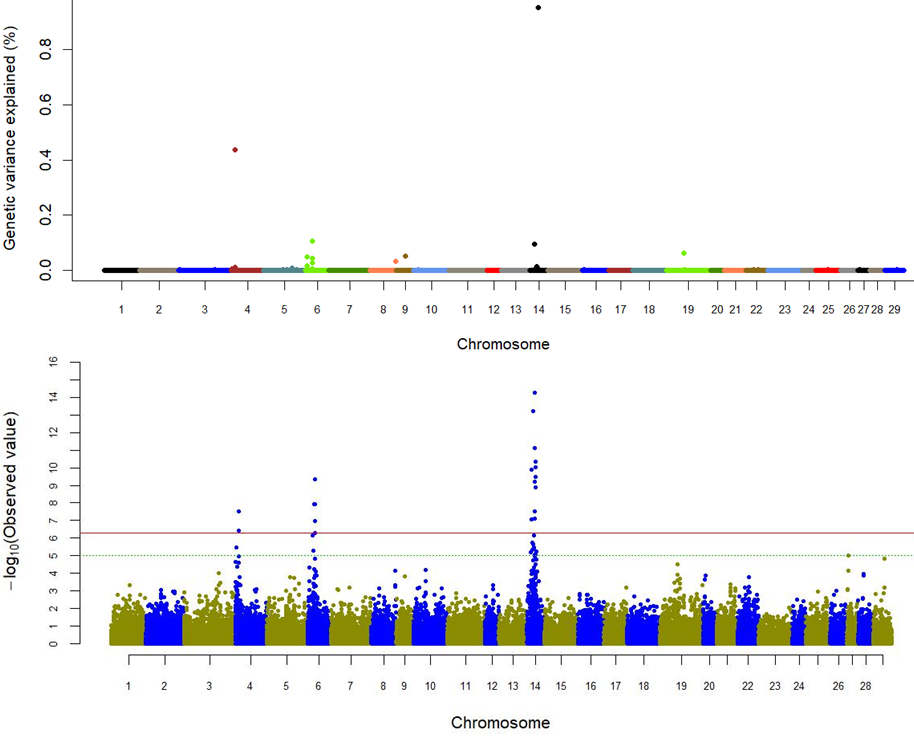


**FIGURE S2 |** Manhattan plot with estimated effects of exon SNPs for CWT in Korean Hanwoo cattle based on BayesR model (up) and GWAS plot based on MLM (below), where Y-axis defines -log10 (P) value against their respective positions on each chromosome (X-axis). The horizontal solid and dot lines indicate the Bonferroni adjusted significant (5.04 × 10^-7^) and suggestive (1.01 × 10^-5^) thresholds level, respectively.


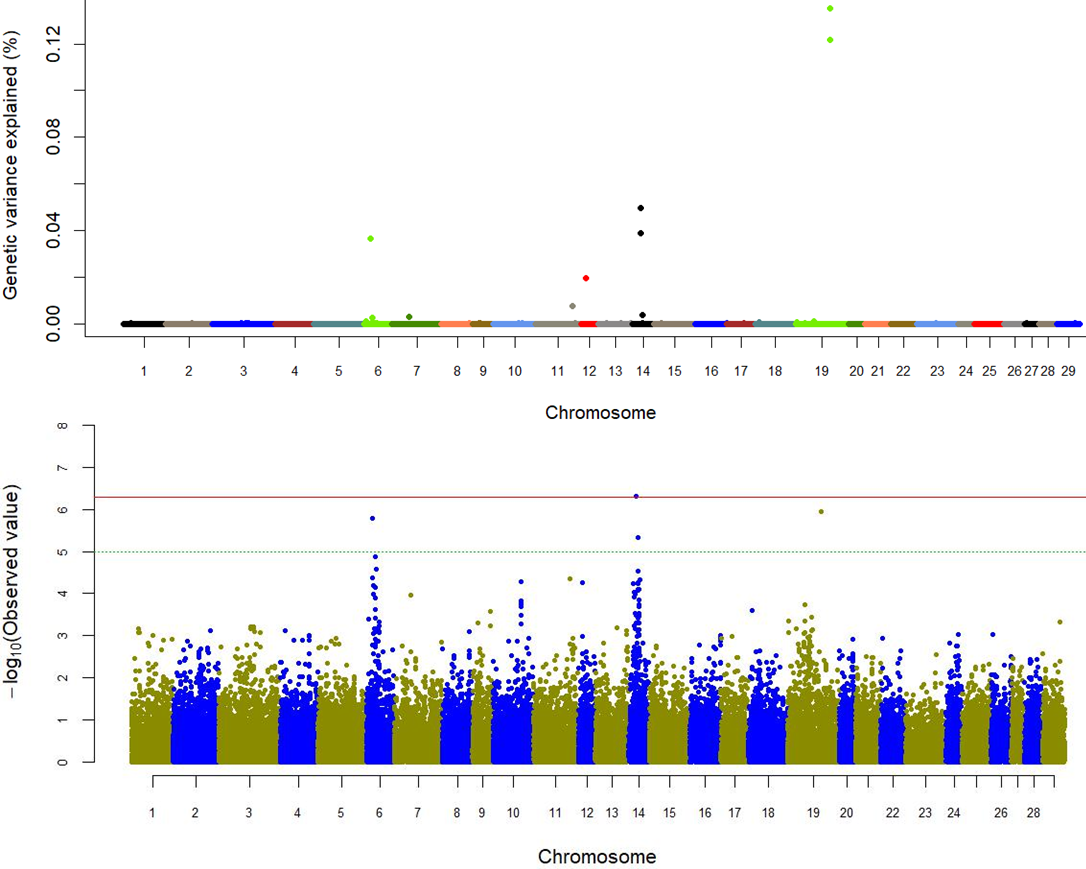


**FIGURE S3 |** Manhattan plot with estimated effects of exon SNPs for EMA in Korean Hanwoo cattle based on BayesR model (up) and GWAS plot based on MLM (below), where Y-axis defines -log10 (P) value against their respective positions on each chromosome (X-axis). The horizontal solid and dot lines indicate the Bonferroni adjusted significant (5.04 × 10^-7^) and suggestive (1.01 × 10^-5^) thresholds level, respectively.


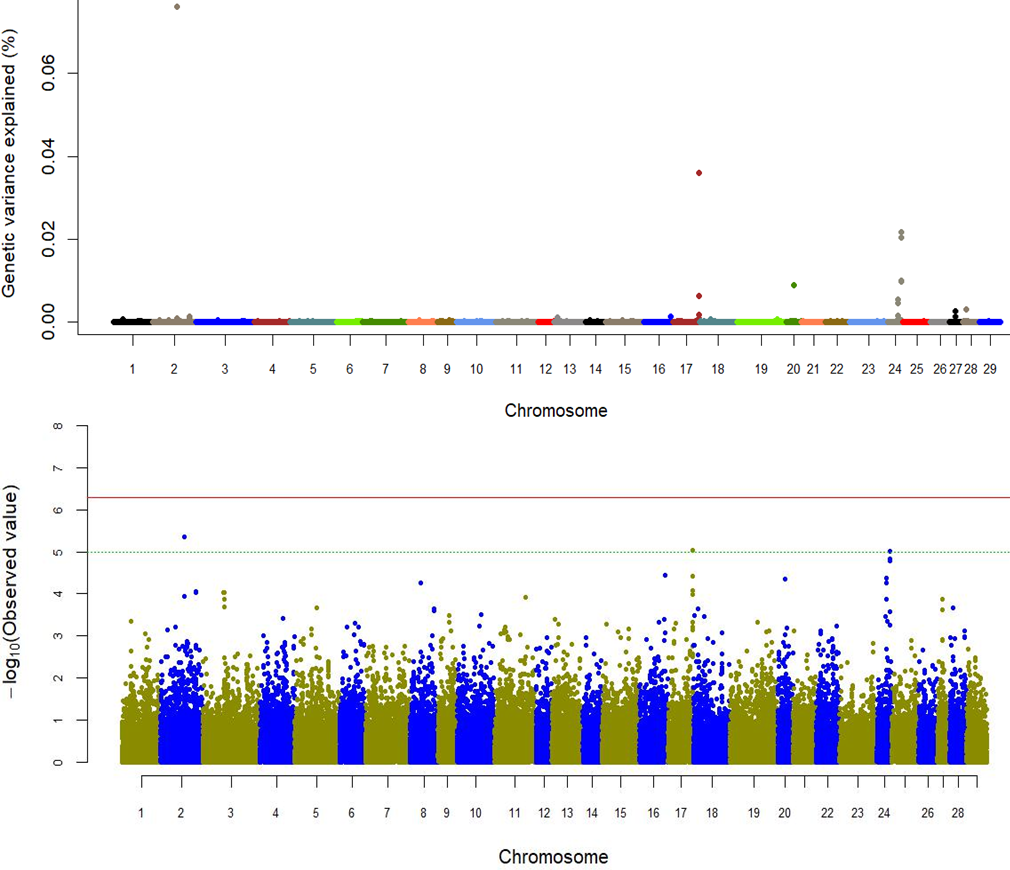


**FIGURE S4 |** Manhattan plot with estimated effects of exon SNPs for BFT in Korean Hanwoo cattle based on BayesR model (up) and GWAS plot based on MLM (below), where Y-axis defines -log10 (P) value against their respective positions on each chromosome (X-axis). The horizontal solid and dot lines indicate the Bonferroni adjusted significant (5.04 × 10^-7^) and suggestive (1.01 × 10^-5^) thresholds level, respectively.


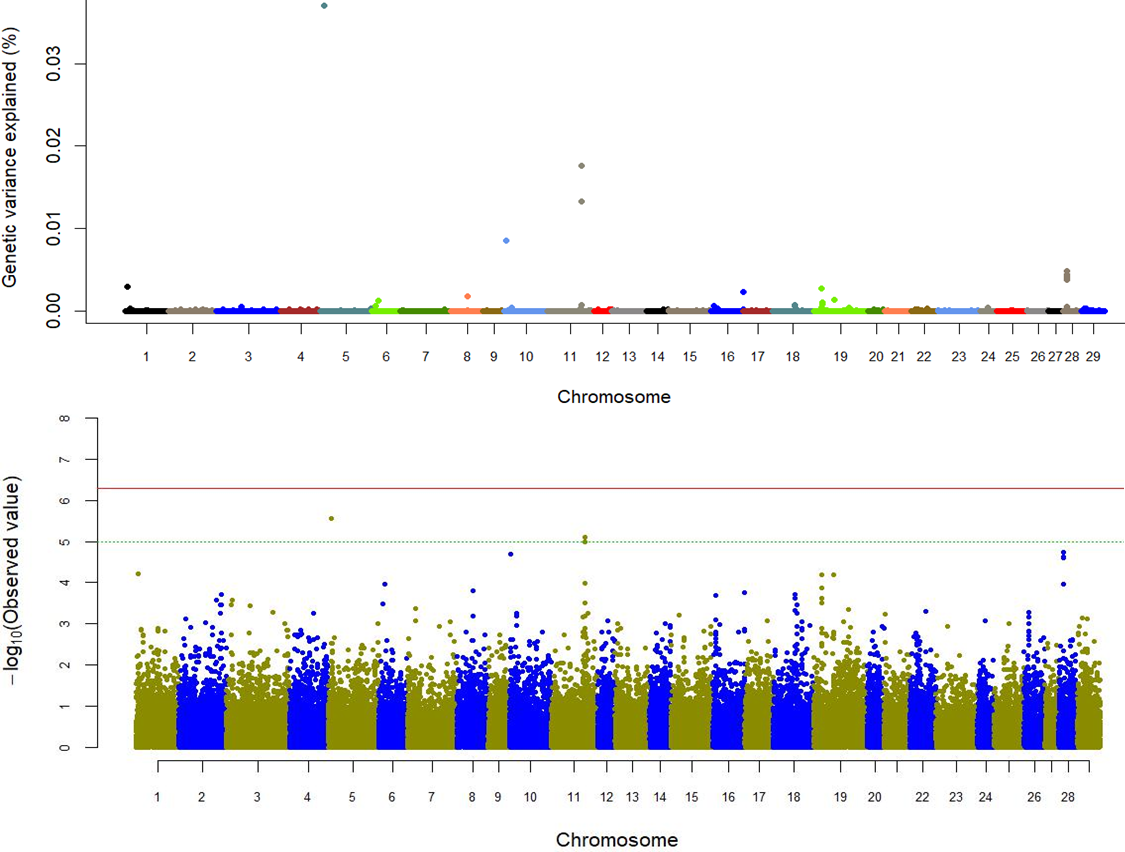


**FIGURE S5 |** Manhattan plot with estimated effects of exon SNPs for MS in Korean Hanwoo cattle based on BayesR model (up) and GWAS plot based on MLM (below), where Y-axis defines -log10 (P) value against their respective positions on each chromosome (X-axis). The horizontal solid and dot lines indicate the Bonferroni adjusted significant (5.04 × 10^-7^) and suggestive (1.01 × 10^-5^) thresholds level, respectively.
